# Different Drugs for the Treatment of Painful Diabetic Peripheral Neuropathy: A Meta-Analysis

**DOI:** 10.3389/fneur.2021.682244

**Published:** 2021-10-29

**Authors:** Lian Jingxuan, Ma Litian, Fu Jianfang

**Affiliations:** ^1^Department of Endocrinology, Xijing Hospital of Air Force Medical University, Xi'an, China; ^2^Department of Gastroenterology, Xijing Hospital of Air Force Medical University, Xi'an, China

**Keywords:** diabetic peripheral neuropathy, painful, meta-analysis, drugs, treatment

## Abstract

**Objective:** To systematically evaluate the effects of different drugs for the treatment of painful diabetic peripheral neuropathy.

**Methods:** All literature from PubMed, Embase, and Cochrane Central Register of Controlled Trials published over the past 12 years (from January 1, 2008 to June 1, 2020) was searched, and two reviewers independently assessed study eligibility, continuous data extraction, independent assessment of bias risk, and graded strength of evidence. The pain score was used as the main result, and 30 and 50% pain reduction and adverse events were used as secondary results.

**Results:** A total of 37 studies were included. Pregabalin, duloxetine, tapentadol, lacosamide, mirogabalin, and capsaicin were all more effective than placebo in alleviating the pain associated with diabetic peripheral neuropathy, while ABT-894 and gabapentin showed no significant effect. In addition, the efficacy of buprenorphine, tanezumab, fulranumab and others could not be concluded due to insufficient studies.

**Conclusion:** Pregabalin and duloxetine showed good therapeutic effects on painful DPN, but adverse events were also significant. The analgesic effects of ABT-894 and gabapentin need to be further studied with longer and larger RCTs. As an opioid drug, tapentadol has a good analgesic effect, but due to its addiction, it needs to be very cautious in clinical use. Although lacosamide, mirogabalin, and capsaicin are more effective than placebo, the therapeutic effect is weaker than pregabalin. For the results of our meta-analysis, long-term studies are still needed to verify their efficacy and safety in the future.

**Systematic Review Registration:** PROSPERO, identifier: CRD42020197397.

## Introduction

Diabetic peripheral neuropathy (DPN) is the most common cause of neuropathy in developed countries, affecting an estimated 50% of people with diabetes. The most common form is chronic, distal, and symmetric sensorimotor polyneuropathy, while other uncommon forms include asymmetric or focal neuropathy, such as diabetic muscle atrophy, trunk radiculopathy, and compression palsy (1). Recent comprehensive reviews of treatments for DPN have been published by the American Association of Neuromuscular and Electrical Diagnostic Medicine, and the American Academy of Neurology; the American Academy of Physical Medicine and Rehabilitation published an article in 2011 demonstrating that pregabalin is an effective treatment method and noted that other treatments for DPN, such as venlafaxine and amitriptyline, may also be effective (2).

The latest systematic review of randomized controlled trials (RCTs) of drug interventions for DPN pain was published in 2017. However, this review did not include some newer drugs and did not incorporate evidence from patients who reported results such as a 30 or 50% pain reduction. Therefore, we present a systematic review of the benefits and disadvantages of drug regimens in relieving DPN pain and health-related quality of life by including the latest randomized controlled trials.

## Methods

### Data Sources and Search Strategy

We performed electronic searches of the following databases: MEDLINE, Embase, and PubMed. We searched each database for nearly 13 years (from January 1, 2008 to June 1, 2020), and the language was limited to English (the complete search strategy is shown in Appendix 1 of the Supplementary Materials). The preferred reporting items of the systematic review and meta-analysis guidelines were followed at all stages of the study (the complete protocol is shown in Appendix 2 of the Supplementary Materials). Our PROSPERO ID is CRD42020197397.

### Inclusion Criteria

We included a double-blind, placebo-controlled RCT of the effects of various analgesics on patients with painful diabetic peripheral neuropathy who were 18 years and older. Studies with an intervention duration <4 weeks or less and extensive pain studies, as well as studies that did not differentiate pDPN patients in the subgroup analysis, were excluded (3). In addition, non-drug treatments such as intravenous injections, physical therapy, over-the-counter drugs and food supplements were excluded. For cross-over RCTs, the carrying effect was taken into account, so we used data from the first phase of the study (4). Our primary outcomes were pain scores (using a validated scale to enhance the reliability of the measurement results) and adverse events. Our secondary outcome was a 30 and 50% pain reduction.

### Data Extraction

Two reviewers independently screened and identified the study and resolved their differences through discussion. In addition, a manual search of references in published systematic reviews and meta-analyses was performed to ensure that no studies were missing. Data were independently extracted to an Excel spreadsheet according to predefined standards. For each of the included studies, we extracted data such as the study time, trial design, intervention measures and time, demographics, and baseline characteristics.

### Data Synthesis and Analysis

The risk of bias was assessed for each included study using the Cochrane Collaboration Risk Assessment tool. For continuous variables, we used the standardized mean difference (SMD) and 95% CI for analysis, and for dichotomous variables, we calculated the risk ratio of the 95% CI. We used changes before and after the intervention to assess the effectiveness of different drugs and placebos. *P* = 0.05 was considered to be statistically significant. Meta-analysis software (RevMan V.5.3) was used for the analyses, heterogeneity was evaluated according to I^2^ = 25%, 50 and 75% values were judged as mild, moderate and substantial heterogeneity, respectively, and heterogeneity was solved by subgroup analysis. GRADE Pro (V.3.6) software was used to rate the overall quality of evidence for each outcome based on five evaluation criteria: risk of bias, inconsistency, indirectness, imprecision, and publication bias.

## Results

**Figure 1:**
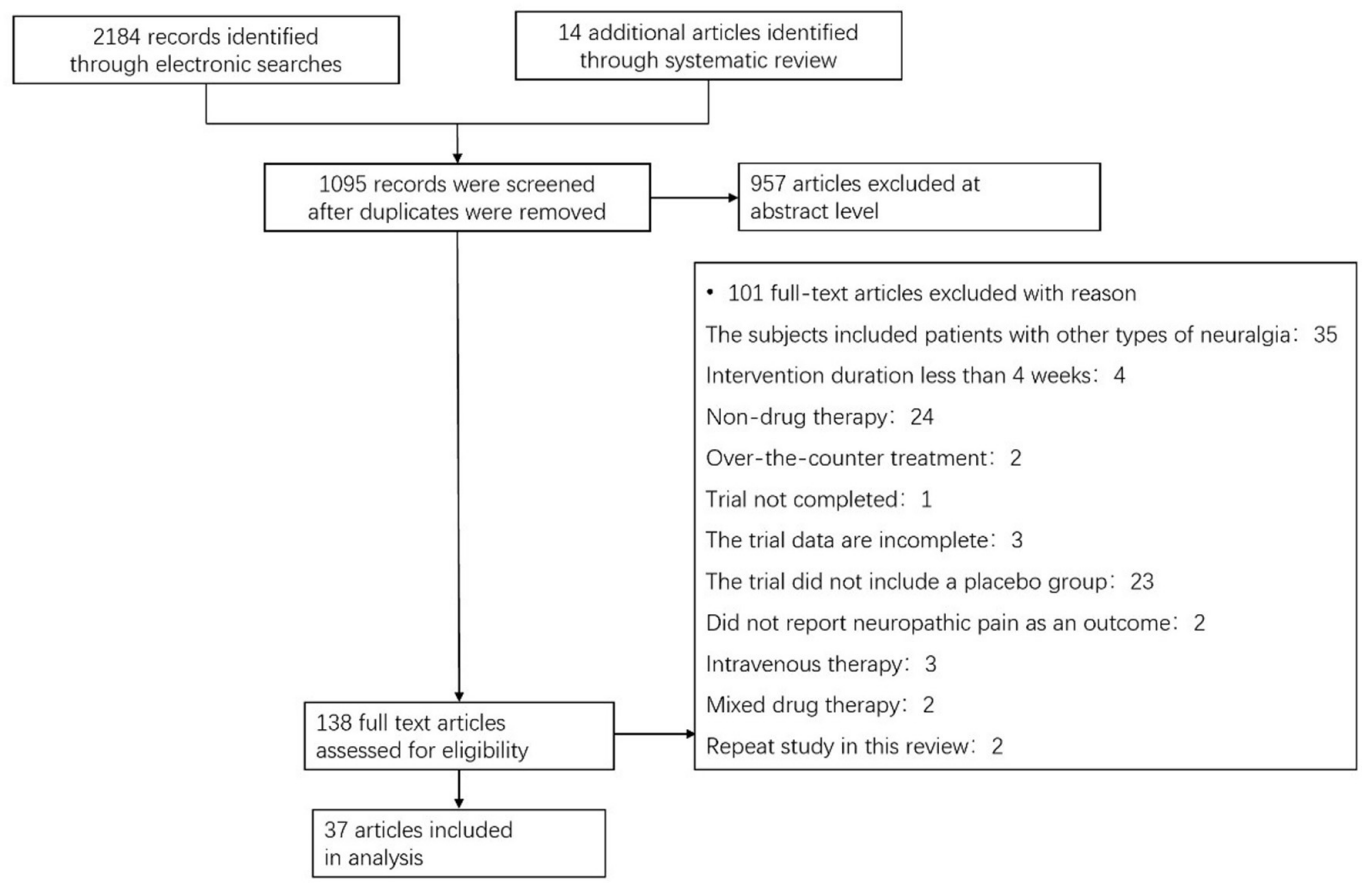
Flow chart showing the process for inclusion of RCTs about diabetic peripheral neuropathy. RCTs, Randomized clinical trials.

A total of 2,184 articles were identified in our search, among which 138 full-text papers were deemed suitable; then, 101 full-text papers were excluded according to the research exclusion criteria. Thirty-seven RCTs on pDPN published between January 1, 2008, and January 1, 2021, met the inclusion criteria (Figure 1) (5–40). Among them, 32.4% evaluated pregabalin, 10.8% evaluated duloxetine, 10.8% evaluated capsaicin, and 8.1% evaluated tapentadol, ABT-894, ABT-594 and clonidine. Treatment lasted from 4 weeks to a year, with most trials were conducted in the US or Europe. Pain outcomes were measured by a numerical rating scale (NRS), visual analog scale (VAS), the Short McGill Pain Questionnaire Visual Assessment Scale (SF-MPQ VAS), and concise pain scale (BPI) (Supplementary Table 1).

Through the risk assessment using the Cochrane Collaboration risk assessment tool, the overall risk was found to be moderate (Supplementary Figures 1, 2), which was mainly caused by allocation concealment, selective reporting, incomplete data, and unclear blind reporting in most studies.

### Pain Score

**Figure 2:**
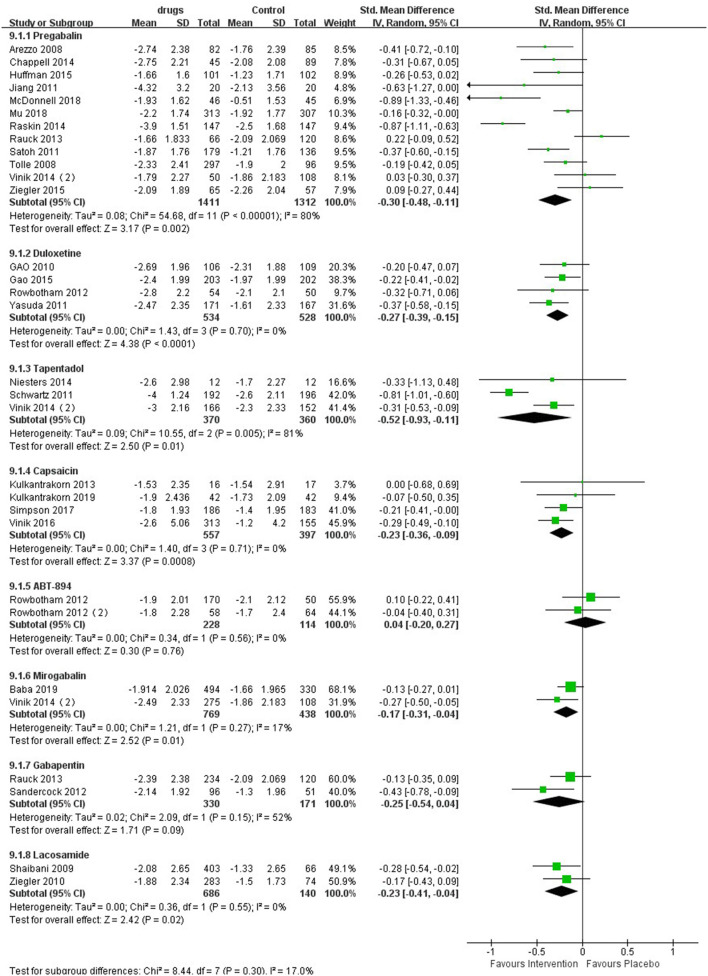
Effects of different drugs on pain scores in patients with diabetic peripheral neuropathy.

As shown in the meta-analysis (Figure 2), compared with patients receiving placebo, those receiving pregabalin [SMD −0.48, −0.11, *P* = 0.002, I^2^ = 80%; Summary of Findings (SoF) Supplementary Table 2] and duloxetine [SMD −0.27 (95% CI −0.39, −0.15, *P* < 0.00001, I^2^ = 0%); SoF Supplementary Table 3], capsaicin [SMD −0.23 (95% CI −0.36, 0.09, *P* < 0.0001, I^2^ = 0%); SoF Supplementary Table 4], tapentadol [SMD −0.52 (95% CI −0.93, 0.11, *P* = 0.01, I^2^ = 81%); SoF Supplementary Table 5], mirogabalin [SMD −0.17 (95% CI −0.31, −0.04, *P* = 0.01, I^2^ = 17%); SoF Supplementary Table 6], and lacosamide [SMD −0.23 (95% CI −0.41, −0.04, *P* = 0.02, I^2^ = 0%); SoF Supplementary Table 7] had significantly lower pain scores. Patients receiving ABT - 894 [SMD 0.04 (95% CI 0.20, 0.27, *p* = 0.76, I^2^ = 0%); SoF Supplementary Table 8] and gabapentin [SMD −0.25 (95% CI −0.54, 0.04, *P* = 0.09, I^2^ = 52%); SoF Supplementary Table 9] had no significant difference in pain scores compared with those receiving placebo (Figure 2). Due to the large number of included studies and high heterogeneity of pregabalin, we conducted a subgroup analysis of pregabalin dose, intervention duration and article quality (Supplementary Figures 3–5). The results showed that pregabalin showed the same direction of effect in terms of drug dose, intervention time and high-quality studies. Only in low-quality studies was no significant difference found. Eleven studies used a NRS to measure pain, and eight showed that patients in the treatment group had a significant reduction in pain scores compared with those in the placebo group. The results of the three studies showed no significant differences between the two groups. In one study, a VAS pain scale was used, and patient pain scores revealed that pregabalin was superior to placebo. For lacosamide, due to different drug dose gradients in the two studies, we performed a subgroup analysis according to drug dose. The pain scores of patients receiving lacosamide were significantly reduced in both low-dose groups [SMD −0.25 (95% CI −0.44, −0.05, *P* = 0.02), I^2^ = 0%] and the high-dose group [SMD −0.20 (95% CI −0.41, 0.00, *P* = 0.05), I^2^ = 0%], with a significant difference compared with the placebo group (Supplementary Figure 6).

### 30% Pain Reduction

**Figure 3:**
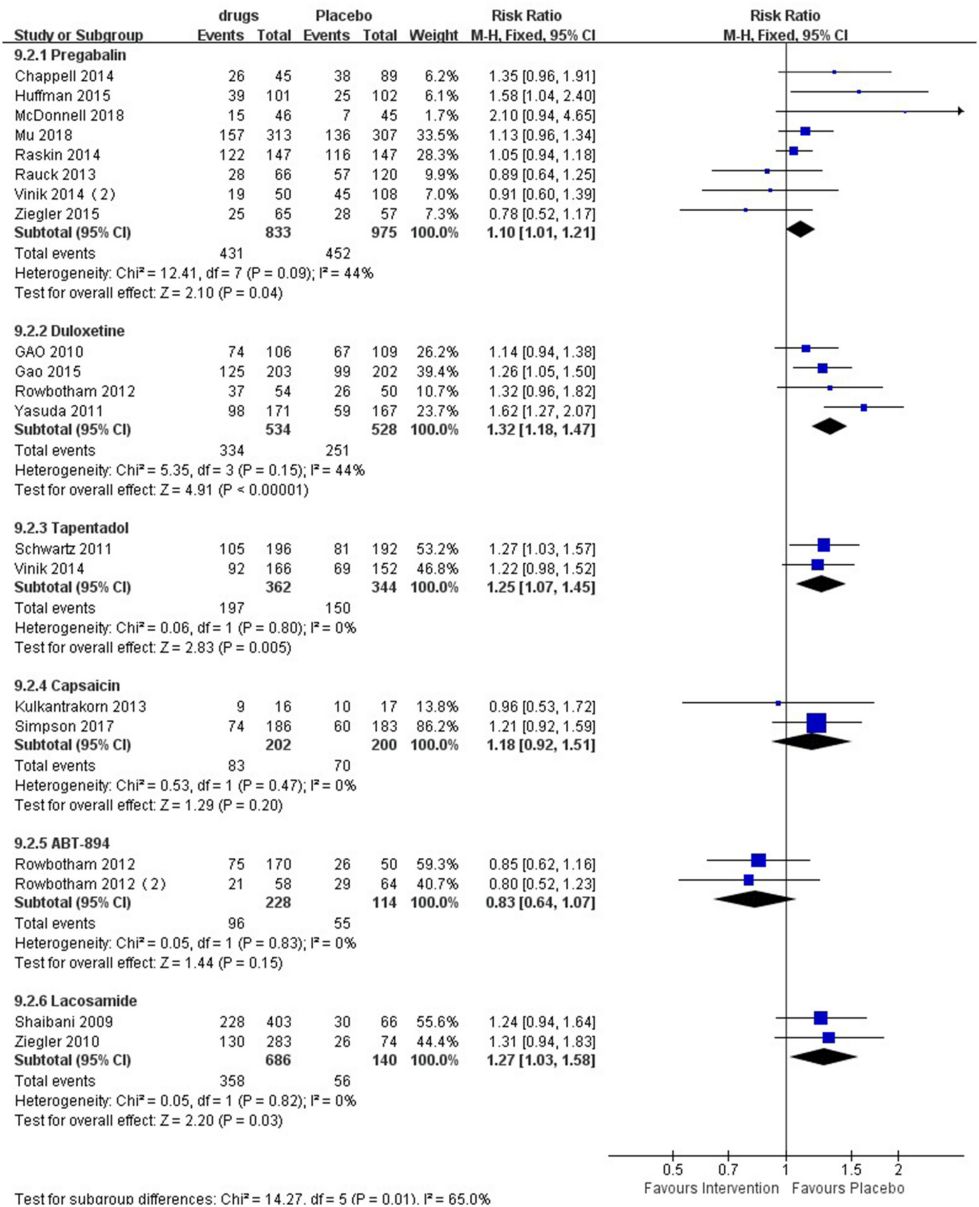
Effect of different drugs on 30% pain reduction in patients with diabetic peripheral neuropathy.

The forest plot (Figure 3) showed that six medicines could elicit 30% pain reduction, among which pregabalin [RR 1.10 (95% CI 1.01, 1.21, *P* = 0.04, I^2^ = 44%; SoF Supplementary Table 2)], duloxetine [RR 1.32 (95% CI 1.18, 1.47, *P* < 0.00001, I^2^ = 44%; SoF Supplementary Table 3)], tapentadol [RR 1.25 (95% CI 1.07, 1.45, *P* = 0.005, I^2^ = 0%; SoF Supplementary Table 5)], and lacosamide [RR 1.27 (95% CI 1.03, 1.58, *P* = 0.03, I^2^ = 0%; SoF Supplementary Table 7)] elicited a significantly higher 30% pain reduction than placebo, while capsaicin [RR 1.18 (95% CI 0.92, 1.51, *P* = 0.20, I^2^ = 0%; SoF Supplementary Table 4)] and ABT-894 [RR 0.83 (95% CI 0.64,1.07, *P* = 0.15, I^2^ = 0%; Supplementary Table 8)] showed no statistically significant difference from placebo. Subgroup analysis of lacosamide showed that there was a significant difference between the high-dose group and the placebo group [RR 1.33 (95% CI 1.06, 1.68, *P* = 0.01, I^2^ = 0%)], while there was no significant difference between the low-dose group and the placebo group [RR 1.22 (95% CI 0.98, 1.53, *P* = 0.08, I^2^ = 0%)] (Supplementary Figure 7). Mirogabalin and gabapentin did not elicit a 30% pain reduction.

### 50% Pain Reduction

**Figure 4:**
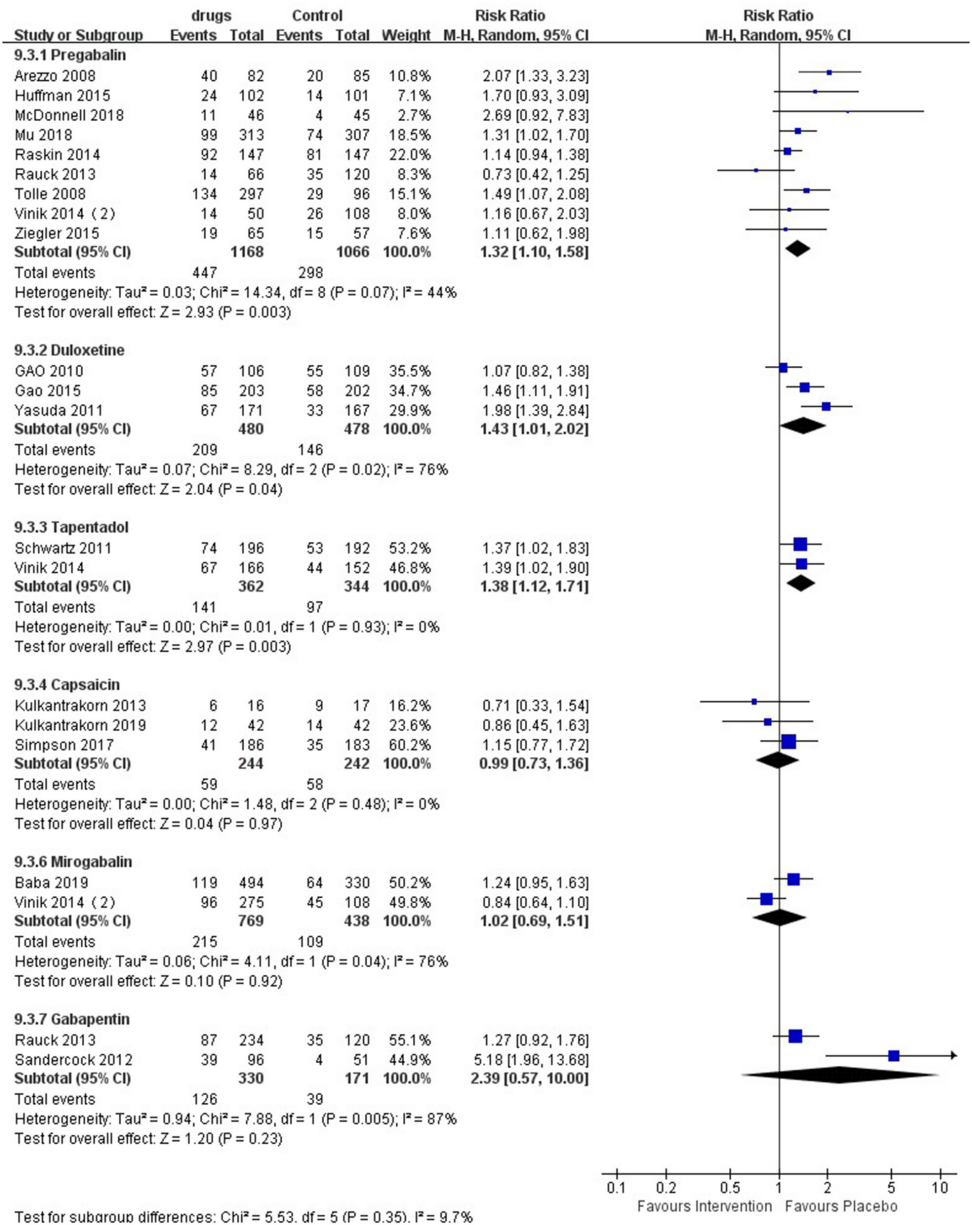
Effect of different drugs on 50% pain reduction in patients with diabetic peripheral neuropathy.

Six medicines could also elicit a 50% pain reduction (Figure 4), among which pregabalin [RR 1.32 (95% CI 1.10, 1.58, *P* = 0.003, I^2^ = 44%; SoF Supplementary Table 2)], duloxetine [RR 1.43 (95% CI 1.01, 2.02, *P* < 0.04, I^2^ = 76%; SoF Supplementary Table 3)], and tapentadol [RR 1.38 (95% CI 1.12,1.71, *P* = 0.003, I^2^ = 0%; SoF Supplementary Table 5)] had a significantly higher 50% pain reduction than placebo, while capsaicin [RR 0.99 (95% CI 0.73,1.36, *P* = 0.97, I^2^ = 0%; SoF Supplementary Table 4)], mirogabalin [RR 1.02 (95% CI 0.69, 1.51, *P* = 0.92, I^2^ = 76%; SoF Supplementary Table 6)], and gabapentin [RR 2.39 (95% CI 0.57, 10.00, *P* = 0.23, I^2^ = 87%; SoF Supplementary Table 9)] showed no significant difference from placebo. ABT-894 with lacosamide did not elicit a 50% pain reduction.

Other agents: In addition, RCTs on tanezumab, tocotrienols, and other agents for the treatment of pDPN, such as Sativex and nabilone, have also been reported. However, due to the small number of studies, we cannot draw firm conclusions regarding their effectiveness.

### Adverse Events

As shown in Table 1, Pregabalin is the most commonly used analgesic. Adverse events such as drowsiness, dizziness, peripheral edema, weight gain, headache, and dizziness have been reported. Among them, drowsiness, dizziness, and peripheral edema are the most common, and almost all studies have reported. Dizziness and drowsiness are most common in duloxetine, mirogabalin, and gabapentin. Headache mainly occurs in ABT-894. Nausea is mainly seen in duloxetine and tapentadol. Pain and burning at the site of application is mainly seen in topical drugs such as capsaicin. Other major adverse events mainly include dizziness and nausea (lacosamide), nausea and constipation (buprenorphine), arthralgia and pain in the extremities (tanezumab), nausea and vomiting (LY545694), arthritis and peripheral edema (fulranumab), constipation (PF-05089771), nausea (venlafaxine and ABT-594), and skin and subcutaneous tissue dysfunction (tocotrienols). No adverse events have been reported for citrullus colocynthis and Sativex. An adverse event was reported for nabilone but it was not specified.

**Table 1:** Effects of different drugs on the risk of adverse events in patients with diabetic peripheral neuropathy.

**Drugs**	**Most frequent adverse events**	**Numbers/total**	**Drugs**	**Most frequent adverse events**	**Numbers/total**
Pregabalin	Somnolence	121/1201	Gabapentin	Nausea	23/330
	Headache	33/688		Somnolence	31/330
	Dizziness	119/1201		Dizziness	47/330
	Nausea	39/754		Muscle spasms	17/234
	Fatigue	33/778	Capsaicin	Application site pain	108/499
	Weight increased	36/584		Burning sensation	71/564
	Edema peripheral	83/999		Application site erythema	33/568
	Diarrhea	20/526		Pain in extremity	39/499
	Constipation	28/816	Tapentadol	Vomiting	34/362
	Muscle spasms	10/182		Nausea	62/362
	Vision blurred	10/309		Diarrhea	27/362
	Asthenia	11/247		Constipation	21/362
	Decreased appetite	11/202		Dizziness	27/362
	Urinary tract infection	15/500		Anxiety	26/362
Duloxetine	Nasopharyngitis	24/171	Lacosamide	Dizziness	37/283
	Dizziness	26/337		Fatigue	27/283
	Somnolence	43/280	Buprenorphine	Nausea	38/89
	Constipation	20/280		Constipation	28/89
	Nausea	46/337	Tanezumab	Arthralgia	7/38
	Diarrhea	17/280		Pain in extremity	4/48
	Fatigue	15/166	LY545694	Nausea	39/139
Mirogabalin	Nasopharyngitis	73/494		Vomiting	27/139
	Somnolence	75/771		Dizziness	19/139
	Dizziness	66/771	Fulranumab	Arthralgia	6/53
	Edema periphera	39/771		Edema peripheral	6/53
	Weight increased	25/771		Diarrhea	5/53
ABT - 894	Headache	27/231	PF-05089771	Constipation	2/44
	Nausea	12/231	Tocotrienols	Injury, poisoning and procedural complications	9/150
	Fatigue	14/231	Nabilone		0/13
	Dizziness	12/231	Citrullus colocynthis		0/30

**Figure 5:**
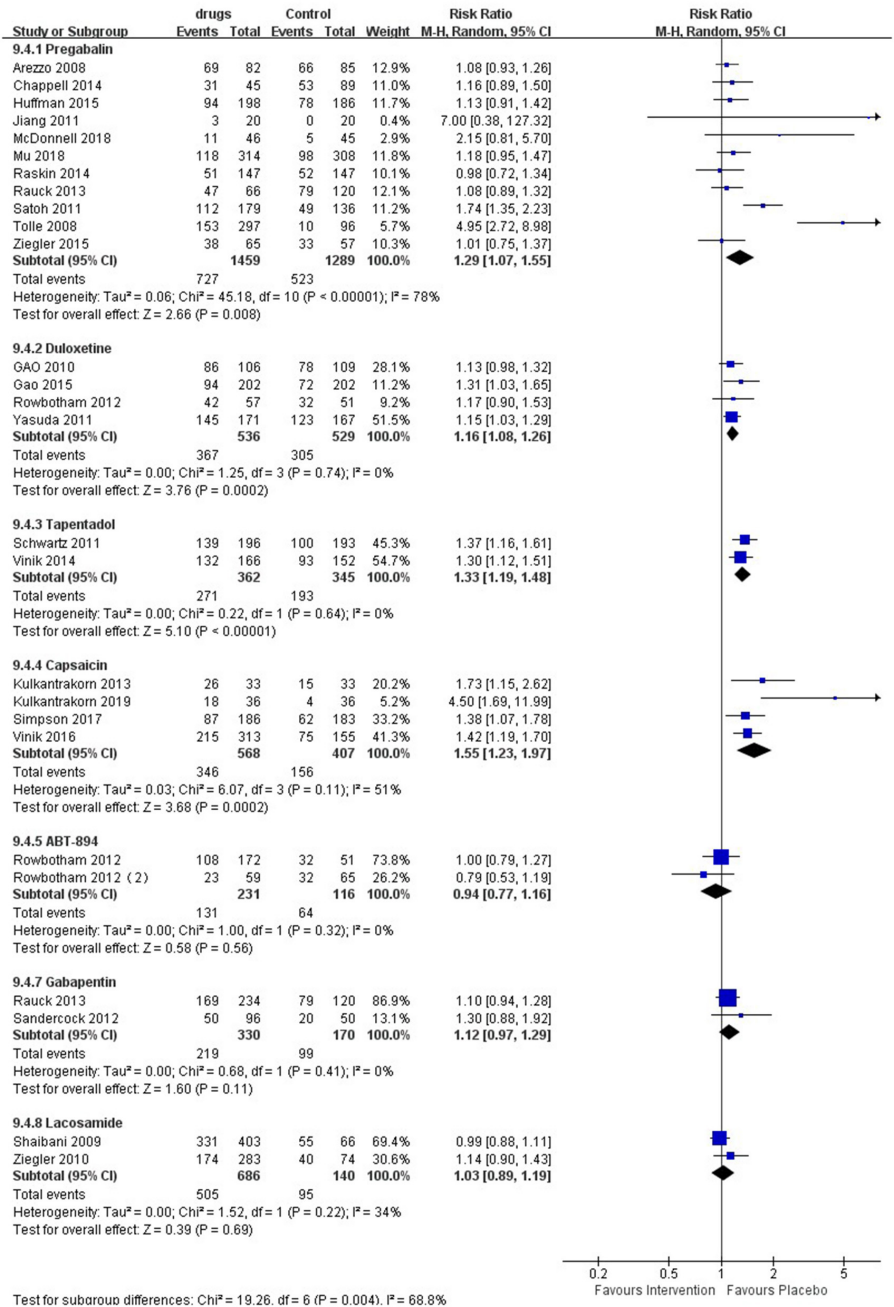
Effects of different drugs on the risk of adverse events in patients with diabetic peripheral neuropathy.

The meta-analysis (Figure 5) showed that patients taking pregabalin [RR 1.29 (95% CI 1.07, 1.55, *P* = 0.008, I^2^ = 78%); SoF Supplementary Table 2], duloxetine [RR 1.16 (95% CI 1.08, 1.26, *P* = 0.00002, I^2^ = 0%); SoF Supplementary Table 3], capsaicin [RR 1.55 (95% CI 1.23, 1.97, *P* = 0.0002, I^2^ = 51%); SoF Supplementary Table 4], and tapentadol [RR 1.33 (95% CI 1.19, 1.48, *P* < 0.00001, I^2^ = 0%); SoF Supplementary Table 5] were more likely to report adverse events than the placebo group. In addition, ABT-894 [RR 0.94 (95% CI, 0.77, 1.16, *p* = 0.56, I^2^ = 0%); SoF Supplementary Table 8], gabapentin (RR 1.12 95% CI 0.97, 1.29, *p* = 0.11, I^2^ = 0%; SoF Supplementary Table 9), and lacosamide (RR 1.03, 95% CI 0.89, 1.19, *P* = 0.69, I^2^ = 34%; SoF Supplementary Table 7) showed no statistically significant difference in terms of the risk of adverse events compared with placebo. The subgroup analysis showed that patients taking pregabalin had a higher risk of adverse events in terms of drug dose (Supplementary Figure 8), longer intervention time (Supplementary Figure 9), and high-quality studies (Supplementary Figure 10). However, in terms of intervention duration <8 weeks and low-quality studies, there was no significant difference between the two groups. Lacosamide showed no significant difference with placebo for either a high dose [RR 1.05 (95% CI 0.96, 1.15, 0 = 0.26, I^2^ = 12%)] or low dose [RR 1.01 (95% CI 0.89, 1.14, *P* = 0.94, I^2^ = 0%)] (Supplementary Figure 11).

## Discussion

### Summary of Evidence

We conducted a meta-analysis based on the available studies published thus far and identified a large amount of related evidence on the effectiveness of different medicines for the treatment of pain in patients with pDPN. Analysis showed that pregabalin, duloxetine, capsaicin, tapentadol and lacosamide were all more effective than placebo, but the quality of evidence was low. Pregabalin, as a first-line clinical drug, is recommended by the guidelines of the European Neurological Association and the American Academy of Neurology (2, 41). Our study showed significant differences in pain scores and 30 and 50% pain reduction compared with placebo, again demonstrating its effectiveness. Due to the large number of included studies and the inconsistency of research methods and pain test standards in different articles, leading to high heterogeneity of pregabalin. Therefore, we used a subgroup analysis to reduce its heterogeneity, and the results of the subgroup analysis showed that pregabalin is more effective than placebo. In addition, study shows that some patients do not tolerate higher doses of pregabalin well. In this case, the therapeutic effect of the lower dose may be insufficient, although dose escalation might be precluded by side effects. In addition, ethnic factors could also play a role (42). Although gabapentin and pregabalin have a similar mechanism of action, the two drugs are often used interchangeably in clinical treatment, but the results of the study show that gabapentin has no significant effect compared with placebo. The newly developed drug ABT894 also showed no significant difference compared with placebo. Although capsaicin and lacosamide showed significant differences in terms of pain scores compared with the placebo, there was no significant difference in their 30% pain reduction compared with placebo. Therefore, the evidence shows that capsaicin and lacosamide could alleviate pain in pDPN patients, but they may not offer a significant degree of relief. In addition, unlike lacosamide, the risk of adverse events is significantly increased with capsaicin. Mirogabalin was developed specifically for the treatment of peripheral neuropathic pain, and our results showed that mirogabalin could significantly reduce the pain score, but there was no significant difference compared with placebo in terms of the 50% pain reduction. Duloxetine is a selective serotonin and norepinephrine reuptake inhibitor that has been widely used in the clinic.

In terms of adverse events, pregabalin, tapentadol, duloxetine, and capsaicin had a higher risk of adverse events than placebo, while lacosamide, gabapentin, and ABT894 had no significant difference. In addition, evidence shows that the risk of adverse events of tapentadol and capsaicin is significantly higher than that of pregabalin and duloxetine. According to studies, the tolerability of pregabalin is equal to or slightly worse than that of placebo in clinical trials (43, 44), which are mainly conducted in North America and/or Europe. The researchers compared the safety data of Western RCTs evaluating pregabalin for painful DPN with data from two similar trials in East Asian origin and found that patients of East Asian origin have more common side effects such as dizziness, drowsiness, peripheral edema and weight gain than whites (45). This may be due to the relatively low average weight of patients of East Asian origin and increased exposure to pregabalin, which may lead to reduced tolerance and may lead to a decrease in the average prescribed dose. On the other hand, although race does not seem to have a significant effect on the pharmacokinetics of pregabalin (44), since the vast majority of clinical trials have been conducted in Europe/North America. More high-quality trials are needed in East Asia to further verify the ethnic differences in the pharmacodynamics. Our research shows that duloxetine is better than pregabalin in terms of 30 and 50% pain reduction. In addition, significant differences have been observed in adverse events. Therefore, we comprehensively consider that duloxetine may have a better effect on painful DPN.

In addition, due to the limitation of the number and quality of RCTs on analgesic drugs, Therefore, the efficacy and safety of all the drugs we analyzed need to be verified by high-quality long-term trials.

We also compared our results with those of several published studies evaluating different drug treatments for painful DPN and found that our results were somewhat consistent with those of other studies. In 2008, a meta-analysis on the effect of pregabalin in the treatment of painful DPN was conducted and revealed that pregabalin was more effective than placebo in the treatment of pain associated with painful DPN due to the early study time, lack of risk assessment and quality of evidence in the included studies (46). Zhang et al. showed that pregabalin was significantly more effective than placebo in treating DPN-related pain, but they did not base their findings on the baseline changes between groups (47). Recently, the therapeutic effect of pregabalin on neuralgia was investigated, and pregabalin was found to have a good effect on patients with peripheral neuralgia, but this study included patients with postherpetic neuralgia (PNH) (48). In addition, these studies only examined the efficacy of pregabalin. Waldfogel et al. analyzed pregabalin, tapentadol and capsaicin that both pregabalin and tapentadol had significant therapeutic effects on painful DPN. However, their study revealed that 0.075% capsaicin showed no significant difference compared with placebo, mainly due to the comprehensive analysis of capsaicin at different concentrations (43).

Due to the short intervention time of most studies, the intervention duration was <3 months; only a small number of studies were more than 3 months. Because painful DPN itself requires long-term treatment, these drugs are often used in the clinic as long-term drugs to relieve symptoms in patients with painful DPN. Therefore, we cannot evaluate the long-term effects or adverse events of these medications. In particular, for opioids, tapentadol was found to have a better therapeutic effect on painful DPN. Our study showed that tapentadol had better pain scores and 30% and 50% pain reduction rates than other medicines; in particular, tapentadol had the greatest reduction in pain scores compared to the placebo group. However, since all of the studies we included were short-term studies, our results showed that tapentadol also significantly increased the risk of adverse events. In addition, the guidelines of the American Academy of Neurology hold that opioids are not recommended for the treatment of chronic pain due to the lack of evidence of long-term efficacy and increasing evidence of the serious risks of opioids, especially addiction and abuse (49).

### Strengths and Limitations of This Study

There are many limitations to our analysis and evidence. We excluded studies with mixed populations of painful DPN and other types of peripheral neuropathy, such as PNH, which excluded some relevant data to an extent. In addition, many studies often use multiple estimates to evaluate pain outcomes, and different studies use different tools to report pain, which may also influence our analysis results. Second, the pain scale itself has many limitations because it can only assess pain at a certain time point and cannot reflect other important aspects of pain treatment, such as improvements in patient function. There are few studies on other drugs, such as hemp drugs, tanezumab, and tocotrienols, so meta-analyses cannot be carried out. Because some studies fail to report specific information on blindness and allocation concealment and provide an incomplete reporting of results, we often downgraded trials in the bias risk assessments.

## Conclusions

In summary, our results suggest that pregabalin, duloxetine and tapentadol have good efficacy in the treatment of DPN pain. These three drugs are also the most common drugs for the clinical treatment of painful DPN at present and are also the three drugs approved by the US Food and Drug Administration (FDA) for its treatment. Lacosamide, milobalin, and capsaicin also have a certain effect compared with placebo, but there is no significant difference between ABT-894 and gabapentin and placebo. However, due to limitations such as fewer RCTs related to these drugs and shorter follow-up time, it is still necessary to design large sample RCTs with strict criteria and long-term follow-up periods to prove the efficacy of these drugs and to better guide clinical decision-making, patient selection and clinical practice guidelines.

## Data Availability Statement

The original contributions presented in the study are included in the article/Supplementary Material, further inquiries can be directed to the corresponding author/s.

## Author Contributions

LJ was mainly involved in electronic retrieval, abstract screening, data extraction, data analysis, and manuscript review, while FJ was mainly involved in method design, electronic retrieval, abstract screening, data extraction, data analysis and interpretation, and manuscript review. ML participated in the revision of the manuscript. All authors contributed to the article and approved the submitted version.

## Funding

This research was funded by the National Natural Science Foundation of China (81670736).

## Conflict of Interest

The authors declare that the research was conducted in the absence of any commercial or financial relationships that could be construed as a potential conflict of interest.

## Publisher's Note

All claims expressed in this article are solely those of the authors and do not necessarily represent those of their affiliated organizations, or those of the publisher, the editors and the reviewers. Any product that may be evaluated in this article, or claim that may be made by its manufacturer, is not guaranteed or endorsed by the publisher.
