# Supplementary material for: Different Drugs for the Treatment of Painful Diabetic Peripheral Neuropathy: A Meta-Analysis
Source: Front Neurol. 2021 Oct 29;12:682244. doi: 10.3389/fneur.2021.682244 (PMC8585758; doi:10.3389/fneur.2021.682244)
Supplement: Supplementary file 1 [file Table_1.DOCX]

Appendix 1: Search strategy for identifying RCTs assessing the effects of different drugs in the treatment of painful diabetic peripheral neuropathy

Medline

1. pain.mp. or Pain

2. pain*.mp.

3. Analgesia

4. Neuralgia

5. 1 or 2 or 3 or 4

6. clinical trials.mp. or Clinical Trial

7. randomized clinical trial.mp.

8. controlled clinical trial.mp. or Controlled Clinical Trial

9. double-blind trial.mp.

10. placebo.ab.

11. ((doubl$ or tripl$ or trebl$) adj5 (blind$ or mask$)).ti,ab.

12. 6 or 7 or 8 or 9 or 10 or 11

13. diabetic

14. 5 and 12 and 13

**Pubmed**

#1 (((("drug therapy"[MeSH Terms] OR ("drug"[All Fields] AND "therapy"[All Fields])) OR "drug therapy"[All Fields]) OR "pharmacotherapies"[All Fields]) OR "drug therapy"[MeSH Subheading]) OR "pharmacotherapy"[All Fields]

#2 (((((((((("diabete"[All Fields] OR "diabetes mellitus"[MeSH Terms]) OR ("diabetes"[All Fields] AND "mellitus"[All Fields])) OR "diabetes mellitus"[All Fields]) OR "diabetes"[All Fields]) OR "diabetes insipidus"[MeSH Terms]) OR ("diabetes"[All Fields] AND "insipidus"[All Fields])) OR "diabetes insipidus"[All Fields]) OR "diabetic"[All Fields]) OR "diabetics"[All Fields]) OR "diabets"[All Fields]) AND (((("peripheral nervous system diseases"[MeSH Terms] OR ((("peripheral"[All Fields] AND "nervous"[All Fields]) AND "system"[All Fields]) AND "diseases"[All Fields])) OR "peripheral nervous system diseases"[All Fields]) OR ("peripheral"[All Fields] AND "neuropathy"[All Fields])) OR "peripheral neuropathy"[All Fields])

#3 ((((((((((("diabete"[All Fields] OR "diabetes mellitus"[MeSH Terms]) OR ("diabetes"[All Fields] AND "mellitus"[All Fields])) OR "diabetes mellitus"[All Fields]) OR "diabetes"[All Fields]) OR "diabetes insipidus"[MeSH Terms]) OR ("diabetes"[All Fields] AND "insipidus"[All Fields])) OR "diabetes insipidus"[All Fields]) OR "diabetic"[All Fields]) OR "diabetics"[All Fields]) OR "diabets"[All Fields]) AND (((("peripheral nervous system diseases"[MeSH Terms] OR ((("peripheral"[All Fields] AND "nervous"[All Fields]) AND "system"[All Fields]) AND "diseases"[All Fields])) OR "peripheral nervous system diseases"[All Fields]) OR ("peripheral"[All Fields] AND "neuropathy"[All Fields])) OR "peripheral neuropathy"[All Fields])) AND ((((("drug therapy"[MeSH Terms] OR ("drug"[All Fields] AND "therapy"[All Fields])) OR "drug therapy"[All Fields]) OR "pharmacotherapies"[All Fields]) OR "drug therapy"[MeSH Subheading]) OR "pharmacotherapy"[All Fields])

**EMBASE**1. pain/ or neuropathic pain

2. analgesi*.mp.

3. 1 or 2

4. diabetic

5. controlled clinical trial/ or randomized clinical trial.mp.

6. double blind procedure

7. placebo*.ab.

8. random*.ab.

9. ((doubl* or trebl* or tripl*) adj25 (blind* or mask*)).ab.

10. 5 or 6 or 7 or 8 or 9

11. 3 and 4 and 10

**Appendix 2: Systematic review protocol**

**Meta-analysis of** **different drugs in the treatment of painful diabetic peripheral neuropathy**

LIAN Jingxuan

Department of Endocrinology, Xijing Hospital, PLA Air Force Military Medical University, Xian, Shanxi, China

Corresponding author: Fu Jianfang, Department of Endocrinology, Xijing Hospital, PLA Air Force Military Medical University, Xian, Shanxi, China

BACKGROUND

Diabetic peripheral neuropathy (DPN) is the most common cause of neuropathy in developed countries, affecting an estimated 50% of people with diabetes; The most common form is chronic, distal, and symmetric sensorimotor polyneuropathy, while other uncommon forms include asymmetric or focal neuropathy, such as diabetic muscle atrophy, trunk radiculopathy, and compression palsy. A recent comprehensive review for the treatment of DPN is by the American association of neuromuscular and electrical diagnostic medicine, the American academy of neurology, the American academy of physical medicine and rehabilitation in 2011 published an article thought Pregabalin is an effective treatment method, and points out the other treatment for DPN may effective medicines such as venlafaxine and amitriptyline.

The latest systematic review of randomized controlled trials (RCTS) of drug interventions for painful DPN was published in 2017. However, the review did not include some newer drugs and did not incorporate evidence from other patients reporting results such as a 30% and 50% pain reduction. Therefore, we present a systematic review of the benefits and disadvantages of drug regimens in relief DPN pain and health-related quality of life by including the latest randomized controlled trials.

**Objective**

To systematically evaluate the effects of different drugs in the treatment of painful diabetic peripheral neuropathy.

**Methods**

We will conduct electronic searches in the following databases: MEDLINE, Embase, Pubmed

We searched each database for nearly 13 years (from January 1, 2008 to January 1, 2020), and the language was limited to English. The preferred reporting items of the systematic review and meta-analysis guidelines were followed at all stages of the study.

We included a double-blind, placebo-controlled RCT of the effects of various analgesics on patients with painful Diabetic peripheral neuropathy 18 years and older. Intervention duration < 4 weeks or smaller studies and extensive pain studies, as well as studies that did not differentiate pDPN patients in the subgroup analysis were excluded. In addition, non-drug treatments such as intravenous injections, physical therapy, over-the-counter drugs and food supplements were excluded. For cross-designed RCTS, the carrying effect is taken into account, so we used data from the first phase of the study. Our primary outcomes were pain scores(using a validated scale to enhance the reliability of the measurement results) and adverse events. Our secondary outcome was a 30%, 50% pain reduction.

**Outcomes**
**Primary outcomes**

pain scores(using a validated scale to enhance the reliability of the measurement results)

adverse events

Secondary outcome

30% pain reduction, 50% pain reduction.

The risk of bias was assessed for each included study using the Cochrane Collaboration Risk Assessment tool, which examines the following domains:

• Method of randomisation;

• Concealment of allocation;

• Blinding of participants and personnel;

• Blinding of outcome assessment;

• Incomplete outcome data;

• Selective reporting;

• Other bias (e.g. industry funding, conflicts of interest, etc)

Two reviewers independently screened and identified the study and resolved their differences through discussion.

**Data extraction:**

We will use a excel spreadsheet to extract relevant data from included studies.Data to be extracted will include:

• The basic characteristics (first author, publication year, country, drug intervention)

• Participants (numbers, medical condition, demographics, etc.)

• Intervention (type of intervention and duration)

• Results (primary and secondary outcome measures, effect size, adverse events)

Two reviewers independently screened and identified the study and resolved their differences through discussion.

**Data analyses**

For continuity variables, we used the standardized mean difference (SMD) and 95%CI for analysis, and for dichotomous variables, we calculated the risk ratio of 95%CI. We used changes before and after the intervention to assess the effectiveness of different drugs and placebos. When SD was not reported in the study, we derived SD from other studies included in meta-analysis. Two review authors will independently enter the data onto RevMan, and will also independently cross-check each other’s entry.

**Subgroup analysis and investigation of heterogeneity**

Meta-analysis software(RevMan V.5.3) was used for analysis, and heterogeneity was evaluated according to I^2^: 25%, 50 and 75% values were judged as mild, moderate and substantial heterogeneity respectively, and the heterogeneity was solved by subgroup analysis. we conducted a subgroup analysis of drug dose, intervention duration (≤8 weeks or >8 weeks) and article quality(IF>3 points or IF≤3).

**Rating the quality of the evidence**

GRADE Pro (V.3.6) software was used to rate the overall quality of evidence for each outcome based on five evaluation criteria: Risk of bias, Inconsistency, Indirectness, Imprecision, and publication bias.

The overall quality of the body of the evidence will rated from high to very low as follows:

• High - Further research is very unlikely to change our confidence in the estimate of effect

• Moderate - Further research is likely to have an important impact on our confidence in the estimate of effect and may change the estimate

• Low - Further research is very likely to have an important impact on our confidence in the estimate of effect and is likely to change the estimate

• Very low - We are very uncertain about the estimate We will use Summary of findings (SOF) tables to present these results.

**Patient public involvement**

Because this study was a meta-analysis, we did not recruit patients or the general public to participate in this study.

**Sources of funding**

This study has no related fund sponsorship at present.

**Conflicts of interest**

This study has no related Conflicts of interest at present.

| **Table s1 Main characteristics of RCTs Included in the Network Meta-Analysis** | | | | | | | | | | | | |  |
| --- | --- | --- | --- | --- | --- | --- | --- | --- | --- | --- | --- | --- | --- |
| **study** | **Drug, Dose** | **settings** | **total N** | **Male** | **Mean Age in years (SD)** | **Study Type and Follow-up weeks** | **Duration of DPN in years(SD)** | **Mean baseline pain(SD) (scale type)** | **30% Reduction** | **50% Reduction** | **Discontinuation** | **Adverse Events** |  |
|  |  |  |  |  |  |  |  |  |  |  |  |  |  |
|  |  |  |  |  |  |  |  |  |  |  |  |  |  |
| Campbell 2012 | placebo | multicenter,USA | 182 | 0.4725 | 57.6 (9.5) | Parallel, 12 weeks | 2.9 (1.3) | 6.4 (1.4) (NRS) | 36/90 | 26/90 | 13/90 | 11/90 |  |
|  | 0.1% topical clonidine |  |  |  | 59.4 (9.9) |  | 3.0 (1.3) | 6.5 (1.5) (NRS) | 43/89 | 31/89 | 9/89 | 3/89 |  |
| Schwartz 2011 | placebo | multicenter,USA, Canada | 395 | 0.5949 | 60.6 (10.56) | Parallel, 12 weeks | 5.8 (5.1) | 7.3 (1.43)(NRS) | 81/192 | 53/192 | 59/193 | 100/193 |  |
|  | Tapentadol |  |  |  | 59.9 (10.68) |  | 5.5 (4.9) |  | 105/196 | 74/196 | 59/196 | 139/196 |  |
| Niesters 2014 | tapentadol | single-center,Netherlands | 24 | 0.5833 | 63(Median) | Parallel, 4 weeks | 6(Median) | 6.5(2.08)(VAS) | NR | NR | 0/12 | NR |  |
|  | placebo |  |  |  | 64(Median) |  | 6.5(Median) | 6.5(2.08)(VAS) | NR | NR | 0/12 | NR |  |
| Vinik 2014 | Placebo | multicenter,USA | 318 | 0.588 | 59.0 (9.00) | Parallel, 12 weeks | NR | 6.8 (1.54)(NRS) | 69/152 | 44/152 | 45/152 | 93/152 |  |
|  | tapentadol |  |  |  | 58.5 (10.63) |  | NR | 6.6 (1.52)(NRS) | 92/166 | 67/166 | 46/166 | 132/166 |  |
| Vinik 2016 | SOC | multicenter, European | 468 | 47.86%% | 59.1(10.3) | Parallel, 52 weeks | 4.4(3.6) | 5.5 (1.3)(BPI) | NR | NR | 27 | 75/155 |  |
|  | Capsaicin 30min + SOC |  |  |  | 61.0(10.3) |  | 4.1(3.7) | 5.6(1.3)(BPI) | NR | NR | 24 | 106/156 |  |
|  | Capsaicin 60min + SOC |  |  |  | 60.9 (10.9) |  | 4.4(3.9) | 5.6(1.4)(BPI) | NR | NR | 29 | 109/157 |  |
| Kulkantrakorn 2019 | placebo | single-center, Thailand | 42 | 0.452 | 65.67(NR) | crossover,8 weeks | 3.20(2.66) | 53.6(20.7)(VAS) | NR | 14/42 | NR | 4/36 |  |
|  | 0.075% capsaicin |  |  |  |  |  |  | 53.3(26.4)(VAS) | NR | 12/42 | NR | 18/36 |  |
| Simpson 2017 | Placebo | multicenter,USA | 369 | 0.5826 | 62.0 (10.8) | Parallel, 12 weeks | 5.7(4.0) | 6.4 (1.5)(NRS) | 60/183 | 35/183 | 8/183 | 62/183 |  |
|  | capsaicin 8% |  |  |  | 63.9 (10.6) |  | 5.8(4.0) | 6.6 (1.4)(NRS) | 74/186 | 41/186 | 9/186 | 87/186 |  |
| Kulkantrakorn 2013 | placebo | single-center, Thailand | 33 | 0.4848 | 57.96(NR) | Parallel, 8 weeks | 4.73(5.13) | 5.0(2.93)(VAS) | 10/17 | 9/17 | 6/17 | 15/33 |  |
|  | 0.025% Capsaicin |  |  |  |  |  |  | 4.41(2.49)(VAS) | 9/16 | 6/16 | 6/16 | 26/33 |  |
| Baba 2019 | placebo | multicenter, Japan | 834 | 0.725 | 61.4(NR) | Parallel, 14 weeks | 3.0(NR) | 58.5(21.45)(VAS) | NR | 64/330 | 25/334 | 74/330 |  |
|  | mirogabalin 15mg/day |  |  |  |  |  | 3.0(NR) | 58(19.43)(VAS) | NR | 34/164 | 12/166 | 58/164 |  |
|  | mirogabalin 20mg/day |  |  |  |  |  | 3.46(NR) | 57.3(20.15)(VAS) | NR | 33/165 | 18/168 | 70/165 |  |
|  | mirogabalin 30mg/day |  |  |  |  |  | 3.0(NR) | 58.7(21.49)(VAS) | NR | 51/165 | 24/166 | 103/165 |  |
| Vinik 2014 2 | placebo | multicenter,USA | 452 | 0.5354 | 60.2 (9.57) | Parallel, 5 weeks | 5.9 (4.61) | 7(1.59)(NRS) | 45/108 | 26/108 | 15/108 | 117 |  |
|  | Mirogabalin 5mg/day |  |  |  | 58.9 (9.85) |  | 4.8 (4.05) | 6.88(1.17)(NRS) | 22/55 | 11/55 | 6/57 | 64 |  |
|  | Mirogabalin 10mg/day |  |  |  | 60.9 (9.92) |  | 4.9 (4.40) | 6.79(1.66)(NRS) | 32/56 | 16/56 | 4/57 | 80 |  |
|  | Mirogabalin 15mg/day |  |  |  | 61.4 (8.70) |  | 7.7 (6.40) | 6.56(1.65)(NRS) | 34/51 | 20/51 | 13/57 | 110 |  |
|  | Mirogabalin 20mg/day |  |  |  | 60.4 (8.59) |  | 5.3 (4.39) | 6.69(1.56)(NRS) | 34/56 | 24/56 | 10/56 | 89 |  |
|  | Mirogabalin 30mg/day |  |  |  | 59.3 (8.54) |  | 6.1 (5.26) | 6.75(1.43)(NRS) | 32/57 | 25/57 | 6/57 | 88 |  |
|  | pregabalin 300 mg/day |  |  |  | 59.5 (9.40) |  | 5.7 (5.00) | 6.63(1.59)(NRS) | 19/50 | 14/50 | 15/56 | 69 |  |
| McDonnell 2018 | Placebo | multicenter,Britain | 135 | 0.6296 | 59.2(NR) | Parallel, 4 weeks | 7.5(NR) | 6.17 (1.16)(NRS) | 7/45 | 4/45 | 4/45 | 5/45 |  |
|  | Pregabalin 300mg/day |  |  |  | 58.1(NR) |  | 7.4(NR) | 6.62 (1.42)(NRS) | 15/46 | 11/46 | 8/46 | 11/46 |  |
|  | PF-05089771 300mg/day |  |  |  | 59.3(NR) |  | 5.3(NR) | 6.35 (1.17)(NRS) | 11/44 | 3/44 | 2/44 | 5/44 |  |
| Mu 2018 | Placebo | multicenter,China | 620 | 0.4726 | 60.9 (9.5) | Parallel, 9 weeks | 2.4(NR) | 6.67 (1.15)(NRS) | 136/307 | 74/307 | 36/309 | 98/308 |  |
|  | Pregabalin 300mg/day |  |  |  | 60.2 (10.3) |  | 2.2(NR) | 6.65 (1.12)(NRS) | 157/313 | 99/313 | 29/314 | 113/314 |  |
| Ziegler 2015 | Placebo | multicenter,German | 194 | 0.5309 | 58.9 (8.60) | Parallel, 6 weeks | 6.1 (5.02) | 6.6 (1.27)(NRS) | 28/57 | 15/57 | 4/62 | 33/57 |  |
|  | ABT-639 200mg/day |  |  |  | 59.5 (8.25) |  | 6.5 (7.94) | 6.4 (1.25)(NRS) | 26/57 | 19/57 | 4/62 | 25/57 |  |
|  | pregabalin 300mg/day |  |  |  | 59.6 (8.75) |  | 5.3 (4.38) | 6.6 (1.26)(NRS) | 25/65 | 19/65 | 9/70 | 38/65 |  |
| Huffman 2015 | Placebo | multicenter,USA | 203 | 0.6502 | 58.4 (9.3) | crossover, 6 weeks | 4.8 (4.6) | 6.52 (1.32)(NRS) | 25/102 | 14/102 | 10/186 | 78/186 |  |
|  | Pregabalin 300mg/day |  |  |  | 59.1 (8.5) |  | 4.7 (4.3) | 6.32 (1.36)(NRS) | 39/101 | 24/101 | 22/198 | 94/198 |  |
| Raskin 2014 | Placebo | multicenter,USA | 294 | 0.5272 | 58.8 (9.2) | Parallel, 13 weeks | 5(NR) | 6.7 (1.3)(NRS) | 116/147 | 81/147 | 34/147 | 52/147 |  |
|  | pregabalin 300 mg/day |  |  |  | 58.3 (10.5) |  | 5.2(NR) | 6.8 (1.2)(NRS) | 122/147 | 92/147 | 23/147 | 51/147 |  |
| Rauck 2013 | placebo | multicenter,USA | 420 | 0.5926 | 60.1(10.63) | Parallel, 14 weeks | NR | 6.49(1.26)(NRS) | 57/120 | 35/120 | 30/120 | 79/120 |  |
|  | Gabapentin 1200 mg/day |  |  |  | 57.5 (10.32) |  | NR | 6.64(1.47)(NRS) | 31/62 | 26/62 | 15/62 | 45/62 |  |
|  | Gabapentin 2400 mg/day |  |  |  | 60.8 (8.97) |  | NR | 6.26(1.22)(NRS) | 25/56 | 15/56 | 19/56 | 38/56 |  |
|  | Gabapentin 3600 mg/day |  |  |  | 57.5 (9.87) |  | NR | 6.48(1.43)(NRS) | 66/116 | 46/116 | 38/116 | 86/116 |  |
|  | Pregabalin 300 mg/day |  |  |  | 57.7(10.59) |  | NR | 6.51(1.27)(NRS) | 28/66 | 14/66 | 19/66 | 47/66 |  |
| Sandercock 2012 | placebo | multicenter,USA | 147 | 0.551 | 58(9.1) | Parallel, 4 weeks | NR | 6.74(1.37)(NRS) | NR | 4/51 | 5/51 | 20/50 |  |
|  | gabapentin 3000mg PM |  |  |  | 58(8.0) |  | NR | 6.71(1.34)(NRS) | NR | 16/46 | 6/46 | 27/46 |  |
|  | gabapentin 1200mg AM/1800 mg PM |  |  |  | 60(7.5) |  | NR | 6.44(1.51)(NRS) | NR | 13/50 | 5/50 | 23/50 |  |
| Chappell 2014 | placebo | multicenter,USA | 273 | 0.568 | 55.32 (10) | Parallel, 5 weeks | NR | 6.64 (1.36)(NRS) | 38/89 | NR | 11/89 | 53/89 |  |
|  | LY5454694 21mg/day |  |  |  | 56.95 (8.35) |  | NR | 6.42 (1.37)(NRS) | 18/42 | NR | 19/43 | 33/43 |  |
|  | LY5454694 49mg/day |  |  |  | 58.59 (7.71) |  | NR | 6.25 (1.54)(NRS) | 21/48 | NR | 13/49 | 43/49 |  |
|  | LY5454694 105mg/day |  |  |  | 56.47 (7.77) |  | NR | 6.42 (1.52)(NRS) | 21/47 | NR | 20/47 | 40/47 |  |
|  | Pregabalin 300mg/day |  |  |  | 56.89 (8.19) |  | NR | 6.55 (1.52)(NRS) | 26/45 | NR | 9/45 | 31/45 |  |
| Jiang 2011 | placebo | single center,China | 40 | 0.625 | 59.65 (12.5) | Parallel, 4 weeks | 55.86 (50.81) | 75.37 (12.90) (VAS) | NR | NR | 6/20 | 0/20 |  |
|  | pregabalin 600mg/day |  |  |  | 55.1 (14.36) |  | 120.74 (138.24) | 70.78 (18.76)(VAS) | NR | NR | 5/20 | 3/20 |  |
| Satoh 2011 | placebo | multicentre,Janpan | 317 | 0.757 | 61.3 (9.6) | Parallel, 13 weeks | 4.2 (3.1) | 6.1 (1.4) (NRS) | NR | 29/136 | 16/136 | 49/136 |  |
|  | pregabalin 300mg/day |  |  |  | 61.3 (10.3) |  | 4.3 (3.6) | 6.0 (1.4)(NRS) | NR | 39/134 | 20/134 | 76/134 |  |
|  | pregabalin 600mg/day |  |  |  | 62.2 (10.3) |  | 4.5 (3.9) | 6.1 (1.3)(NRS) | NR | 16/45 | 13/45 | 36/45 |  |
| Gao 2015 | Placebo | multicenter,China | 405 | 0.449 | 61.2 (9.4) | Parallel, 12 weeks | 3.1 (3.1) | 5.6 (1.7)(NRS) | 99/202 | 58/202 | 26/202 | 72/202 |  |
|  | duloxetine 60mg/day |  |  |  | 61.6 (9.7) |  | 3.5 (3.9) | 5.7 (1.7)(NRS) | 125/203 | 85/203 | 30/203 | 94/202 |  |
| Rowbotham 2012 | placebo | multicenter,USA | 280 | 0.5607 | 59.6 (7.0) | Parallel, 8 weeks | 4.4 (3.7) | 6.62 (1.23)(NRS) | 26/50 | NR | NR | 32/51 |  |
|  | ABT-894 1mg/day |  |  |  | 57.5 (9.2) |  | 4.5 (3.4) | 6.76 (1.51)(NRS) | 26/60 | NR | NR | 41/61 |  |
|  | ABT-894 2mg/day |  |  |  | 57.9 (8.0) |  | 4.9 (4.7) | 6.43 (1.43)(NRS) | 24/56 | NR | NR | 35/56 |  |
|  | ABT-894 4mg/day |  |  |  | 58.3 (8.1) |  | 4.8 (4.6) | 6.23 (1.35) (NRS) | 25/54 | NR | NR | 32/55 |  |
|  | Duloxetine 60mg/day |  |  |  | 60.1 (7.8) |  | 4.8 (5.0) | 6.61 (1.37)(NRS) | 37/54 | NR | NR | 42/57 |  |
| Rowbotham 2012 1 | placebo | multicenter,USA | 124 | 0.5645 | 56.5 (10.8) | Parallel, 8 weeks | 5.3 (4.8) | 6.79 (1.58) (NRS) | 29/64 | NR | NR | 32/65 |  |
|  | ABT-894 6mg/day |  |  |  | 57.0 (8.8) |  | 4.2 (3.2) | 6.74 (1.51)(NRS) | 21/58 | NR | NR | 23/59 |  |
| Yasuda 2011 | placebo | multicentre,Janpan | 339 | 0.757 | 60.8(9.2) | Parallel, 12 weeks | 4.2(4.4) | 5.78(1.17)(NRS) | 59/167 | 33/167 | 17/167 | 123/167 |  |
|  | duloxetine 40mg/day |  |  |  | 62.1(9.3) |  | 4.6(3.9) | 5.79(1.23)(NRS) | 47/85 | 32/85 | 13/85 | 72/85 |  |
|  | duloxetine 60mg/day |  |  |  | 59.7(12.1) |  | 4.2(3.7) | 5.76(1.17)(NRS) | 51/86 | 35/86 | 16/86 | 73/86 |  |
| GAO 2010 | placebo | multicenter,China | 215 | 0.4698 | 59.9(9.5) | Parallel, 12 weeks | 3.3(3.4) | 5.5(1.4)(BPI) | 67/109 | 55/109 | 17/109 | 78/109 |  |
|  | Duloxetine 60–120mg/day |  |  |  | 58.6(10.4) |  | 3.1(3.1) | 5.5(1.3)(BPI) | 74/106 | 57/106 | 16/106 | 86/106 |  |
| Candace 2015 | Tanezumab 20mg /day | multicenter,USA | 73 | 0.6438 | 61.6(NR) | Parallel, 8 weeks | 4.6(NR) | 6.6 (1.4)(NRS) | 15/38 | 12/38 | 2/38 | 24/38 |  |
|  | placebo |  |  |  | 59.6(NR) |  | 4.6(NR) | 6.9 (1.5)(NRS) | 6/35 | 4/35 | 1/35 | 19/35 |  |
| Wang 2014 | placebo | multicenter,USA | 77 | 0.558 | 58.0 (9.47) | Parallel, 12 weeks | NR | 7.1 (1.33)(NRS) | NR | 2/24 | 6/24 | 18/24 |  |
|  | Fulranumab 1mg/day |  |  |  | 57.9 (7.15) |  | NR | 6.7 (1.52)(NRS) | NR | 3/16 | 3/16 | 13/16 |  |
|  | Fulranumab 3mg/day |  |  |  | 57.1 (11.36) |  | NR | 7.1 (1.75)(NRS) | NR | 4/14 | 3/14 | 12/14 |  |
|  | Fulranumab 10mg/day |  |  |  | 60.9 (9.90) |  | NR | 7.1 (1.25)(NRS) | NR | 7/23 | 3/23 | 19/23 |  |
| Toth 2012 | placebo | single-center,Canada | 26 | 0.5385 | 61.6(14.6) | Parallel, 4 weeks | 7.2(8.5) | 6.59(2.03)(VAS) | 5/13 | 1/13 | 1/13 | 6/13 |  |
|  | nabilone |  |  |  | 60.8(15.3) |  | 7.1(7.9) | 6.54(1.91)(VAS) | 11/13 | 4/13 | 0/13 | 7/13 |  |
| Selvarajah 2010 | placebo | single-center,Britain | 30 | 0.6333 | 54.4(11.6) | Parallel, 10 weeks | NR | 63.6(14.0)(VAS) | 9/14 | NR | NR | NR |  |
|  | Sativex |  |  |  | 58.2(8.8) |  | NR | 67.1(19.4)(VAS) | 8/15 | NR | NR | NR |  |
| Simpson 2016 | Placebo | multicenter,Australia | 186 | 0.667 | 63.3 (9.3) | Parallel, 12 weeks | NR | 5.9 (1.3)(NRS) | 38/92 | 19/92 | 24/93 | 76/93 |  |
|  | buprenorphine |  |  |  | 62.6 (9.6) |  | NR | 5.7 (1.1)(NRS) | 46/89 | 31/89 | 37/93 | 87/93 |  |
| Heydari 2016 | Placebo | single-center,China | 60 | 0.6833 | 52.70 (10.53) | Parallel, 12 weeks | 1.49(1.31) | 6.43(0.51)(NRS) | NR | NR | 3/30 | 0/30 |  |
|  | Citrullus colocynthis |  |  |  | 57.36 (10.00) |  | 1.58(1.45) | 6.66(0.57)(NRS) | NR | NR | 2/30 | 0/30 |  |
| Hor 2018 | placebo | multicenter,Malaysia | 300 | 0.433 | 57.2(8.9) | Parallel, 52weeks | NR | 7.6 (2.5)(TSS) | NR | NR | 32/150 | 55/150 |  |
|  | tocotrienols 200mg/day |  |  |  | 58(8.9) |  | NR | 8.1 (2.9)(TSS) | NR | NR | 39/150 | 62/150 |  |
| Arezzo 2008 | Placebo | multicenter,USA | 167 | 0.6168 | 58.3 (10.9) | Parallel, 13 weeks | 4.4 (3.7) | 6.58 (1.58)(NRS) | NR | 20/85 | 13/85 | 66/85 |  |
|  | Pregabalin 600mg/day |  |  |  | 58.2 (9.6) |  | 4.9 (3.4) | 6.28 (1.47)(NRS) | NR | 40/82 | 15/82 | 69/82 |  |
| Shaibani 2009 | Placebo | multicenter,USA | 469 | 0.565 | 59.5 (8.3) | Parallel, 18 weeks | 3.1 (1.6) | 6.2 (1.6)(NRS) | 30/66 | 18/66 | 21/66 | 55/66 |  |
|  | Lacosamide 200mg/day |  |  |  | 60.2 (11.1) |  | 3.0 (1.6) | 6.3 (1.5)(NRS) | 76/141 | 38/141 | 46/141 | 113/141 |  |
|  | Lacosamide 400mg/day |  |  |  | 60.3 (9.9) |  | 3.2 (1.5) | 6.4 (1.5)(NRS) | 73/125 | 55/125 | 54/125 | 99/125 |  |
|  | Lacosamide 600mg/day |  |  |  | 59.1 (9.8) |  | 3.0 (1.5) | 6.3 (1.4)(NRS) | 79/137 | 41/137 | 91/137 | 119137 |  |
| Ziegler 2010 | placebo | multicenter,European | 357 | 0.515 | 58.3 (10.0) | Parallel, 18 weeks | 3.0 (2.2) | 6.6 (1.5) (NRS) | 26/74 | NR | 15/74 | 40/74 |  |
|  | Lacosamide 400 mg/day |  |  |  | 58.6 (10.5) |  | 3.3 (2.8) | 6.4 (1.3) (NRS) | 64/150 | NR | 37/150 | 88/150 |  |
|  | Lacosamide 600 mg/day |  |  |  | 57.0 (11.0) |  | 3.3 (2.7) | 6.4 (1.4) (NRS) | 66/133 | NR | 59/133 | 86/133 |  |
| Rowbotham 2009 | Placebo | multicenter,USA | 266 | 0.5451 | 60.2 (11.43) | Parallel, 8 weeks | NR | 6.5 (1.43)(NRS) | NR | NR | 14/66 | 43/66 |  |
|  | ABT-594 150µg/day |  |  |  | 60.8 (10.78) |  | NR | 6.6 (1.69)(NRS) | NR | NR | 25/65 | 54/65 |  |
|  | ABT-594 225µg/day |  |  |  | 61.8 (11.80) |  | NR | 6.7 (1.51)(NRS) | NR | NR | 39/69 | 62/69 |  |
|  | ABT-594 300µg/day |  |  |  | 64.7 (11.10) |  | NR | 6.7 (1.74)(NRS) | NR | NR | 50/67 | 61/67 |  |
| Kadiroglu 2008 | Placebo | single-center,Turkey | 60 | 0.2 | 54.1(10.6) | Parallel, 8 weeks | NR | 7.4(0.8)(NRS) | NR | NR | 0/30 | 0/30 |  |
|  | Venlafaxine |  |  |  | 52.3(7.3) |  | NR | 7.2(1.1)(NRS) | NR | NR | 0/30 | 10/30 |  |
| Tolle 2008 | Placebo | multicenter,Europe | 395 | 0.554 | 58.93 (11.7) | Parallel, 12 weeks | NR | 6.4(2.1)(NRS) | NR | 29/96 | 17/96 | 10/96 |  |
|  | Pregabalin 150mg/day |  |  |  | 58.51 (12.4) |  | NR | 6.2(2.5)(NRS) | NR | 34/99 | 17/99 | 34/99 |  |
|  | Pregabalin 300mg/day |  |  |  | 57.28 (10.5) |  | NR | 6.4(2.2)(NRS) | NR | 33/99 | 20/99 | 58/99 |  |
|  | Pregabalin 600mg/day |  |  |  | 59.70 (11.3) |  | NR | 6.6(2)(NRS) | NR | 46/101 | 23/99 | 61/99 |  |

| **Table s2 Pregabalin compared to placebo for Painful Diabetic Peripheral Neuropathy** | | | | | | |
| --- | --- | --- | --- | --- | --- | --- |
| **Outcomes** | **Illustrative comparative risks* (95% CI)** | | **Relative effect (95% CI)** | **No of Participants (studies)** | **Quality of the evidence (GRADE)** | **Comments** |
|  | Assumed risk | Corresponding risk |  |  |  |  |
|  | **Placebo** | **Pregabalin** |  |  |  |  |
| **studies comparing Pregabalin with placebo for pain outcome** Scale from: 0 to 10. |  | The mean studies comparing Pregabalin with placebo for pain outcome in the intervention groups was **0.3 standard deviations lower** (0.48 to 0.11 lower) |  | 2723 (12 studies) | ⊕⊝⊝⊝ **very low**^1,2,3,4^ | SMD -0.3 (-0.48 to -0.11) |
| **studies comparing Pregabalin with placebo for 30% pain reduction** | **Study population** | | **RR 1.1**  (1.01 to 1.21) | 1808 (8 studies) | ⊕⊕⊝⊝ **low**^1,2,4^ |  |
|  | **464 per 1000** | **510 per 1000** (468 to 561) |  |  |  |  |
|  | **Moderate** | |  |  |  |  |
|  | **435 per 1000** | **479 per 1000** (439 to 526) |  |  |  |  |
| **studies comparing Pregabalin with placebo for 50% pain reduction** | **Study population** | | **RR 1.31**  (1.16 to 1.48) | 2234 (9 studies) | ⊕⊝⊝⊝ **very low**^1,2,3,4^ |  |
|  | **280 per 1000** | **366 per 1000** (324 to 414) |  |  |  |  |
|  | **Moderate** | |  |  |  |  |
|  | **241 per 1000** | **316 per 1000** (280 to 357) |  |  |  |  |
| **Effect of Pregabalin on the risk of adverse events** | **Study population** | | **RR 1.29**  (1.07 to 1.55) | 2748 (11 studies) | ⊕⊝⊝⊝ **very low**^1,3,4^ |  |
|  | **406 per 1000** | **523 per 1000** (434 to 629) |  |  |  |  |
|  | **Moderate** | |  |  |  |  |
|  | **360 per 1000** | **464 per 1000** (385 to 558) |  |  |  |  |
| GRADE Working Group grades of evidence **High quality:** Further research is very unlikely to change our confidence in the estimate of effect.  **Moderate quality:** Further research is likely to have an important impact on our confidence in the estimate of effect and may change the estimate. **Low quality:** Further research is very likely to have an important impact on our confidence in the estimate of effect and is likely to change the estimate. **Very low quality:** We are very uncertain about the estimate. | | | | | | |
| ^1^ Part of the study allocation is hidden without explanation ^2^ Some specific random methods and blind methods are not described ^3^ Study heterogeneity is high ^4^ Some of the experiments were sponsored by related enterprises | | | | | | |

| **Table s3 Duloxetine compared to placebo for Painful Diabetic Peripheral Neuropathy** | | | | | | |
| --- | --- | --- | --- | --- | --- | --- |
| **Outcomes** | **Illustrative comparative risks* (95% CI)** | | **Relative effect (95% CI)** | **No of Participants (studies)** | **Quality of the evidence (GRADE)** | **Comments** |
|  | Assumed risk | Corresponding risk |  |  |  |  |
|  | **Placebo** | **Duloxetine** |  |  |  |  |
| **studies comparing Duloxetine with placebo for pain outcome** |  | The mean studies comparing duloxetine with placebo for pain outcome in the intervention groups was **0.27 standard deviations lower** (0.39 to 0.15 lower) |  | 1062 (4 studies) | ⊕⊕⊝⊝ **low**^1,2^ | SMD -0.27 (-0.39 to -0.15) |
| **studies comparing Duloxetine with placebo for 30% pain reduction** | **Study population** | | **RR 1.32**  (1.18 to 1.47) | 1062 (4 studies) | ⊕⊕⊝⊝ **low**^1,2^ |  |
|  | **475 per 1000** | **628 per 1000** (561 to 699) |  |  |  |  |
|  | **Moderate** | |  |  |  |  |
|  | **505 per 1000** | **667 per 1000** (596 to 742) |  |  |  |  |
| **studies comparing Duloxetine with placebo for 50% pain reduction** | **Study population** | | **RR 1.43**  (1.01 to 2.02) | 958 (3 studies) | ⊕⊝⊝⊝ **very low**^1,2,3^ |  |
|  | **305 per 1000** | **437 per 1000** (308 to 617) |  |  |  |  |
|  | **Moderate** | |  |  |  |  |
|  | **287 per 1000** | **410 per 1000** (290 to 580) |  |  |  |  |
| **Effect of Duloxetine on the risk of adverse events** | **Study population** | | **RR 1.19**  (1.09 to 1.29) | 1065 (4 studies) | ⊕⊕⊝⊝ **low**^1,2^ |  |
|  | **577 per 1000** | **686 per 1000** (628 to 744) |  |  |  |  |
|  | **Moderate** | |  |  |  |  |
|  | **672 per 1000** | **800 per 1000** (732 to 867) |  |  |  |  |
| GRADE Working Group grades of evidence **High quality:** Further research is very unlikely to change our confidence in the estimate of effect.  **Moderate quality:** Further research is likely to have an important impact on our confidence in the estimate of effect and may change the estimate. **Low quality:** Further research is very likely to have an important impact on our confidence in the estimate of effect and is likely to change the estimate. **Very low quality:** We are very uncertain about the estimate. | | | | | | |
| ^1^ Part of the study was blind, random, and allocative hiding ^2^ Part of the study was sponsored by the relevant enterprises ^3^ High heterogeneity | | | | | | |

| **Table s4 Capsaicin compared to Placebo for Painful Diabetic Peripheral Neuropathy** | | | | | | |
| --- | --- | --- | --- | --- | --- | --- |
| **Outcomes** | **Illustrative comparative risks* (95% CI)** | | **Relative effect (95% CI)** | **No of Participants (studies)** | **Quality of the evidence (GRADE)** | **Comments** |
|  | Assumed risk | Corresponding risk |  |  |  |  |
|  | **Placebo** | **Capsaicin** |  |  |  |  |
| **studies comparing Capsaicin with placebo for pain outcome** |  | The mean studies comparing capsaicin with placebo for pain outcome in the intervention groups was **0.23 standard deviations lower** (0.36 to 0.09 lower) |  | 954 (4 studies) | ⊕⊕⊝⊝ **low**^1,2^ | SMD -0.23 (-0.36 to -0.09) |
| **studies comparing Capsaicin with placebo for30% pain reduction** | **Study population** | | **RR 1.18**  (0.92 to 1.51) | 402 (2 studies) | ⊕⊝⊝⊝ **very low**^1,2,3^ |  |
|  | **350 per 1000** | **413 per 1000** (322 to 528) |  |  |  |  |
|  | **Moderate** | |  |  |  |  |
|  | **458 per 1000** | **540 per 1000** (421 to 692) |  |  |  |  |
| **studies comparing Capsaicin with placebo for 50% pain reduction** | **Study population** | | **RR 1.01**  (0.74 to 1.39) | 486 (3 studies) | ⊕⊝⊝⊝ **very low**^1,2,3^ |  |
|  | **240 per 1000** | **242 per 1000** (177 to 333) |  |  |  |  |
|  | **Moderate** | |  |  |  |  |
|  | **333 per 1000** | **336 per 1000** (246 to 463) |  |  |  |  |
| **Effect of Capsaicin on the risk of adverse events** | **Study population** | | **RR 1.55**  (1.23 to 1.97) | 975 (4 studies) | ⊕⊝⊝⊝ **very low**^1,2,4^ |  |
|  | **383 per 1000** | **594 per 1000** (471 to 755) |  |  |  |  |
|  | **Moderate** | |  |  |  |  |
|  | **397 per 1000** | **615 per 1000** (488 to 782) |  |  |  |  |
| GRADE Working Group grades of evidence **High quality:** Further research is very unlikely to change our confidence in the estimate of effect.  **Moderate quality:** Further research is likely to have an important impact on our confidence in the estimate of effect and may change the estimate. **Low quality:** Further research is very likely to have an important impact on our confidence in the estimate of effect and is likely to change the estimate. **Very low quality:** We are very uncertain about the estimate. | | | | | | |
| ^1^ Some studies on blind method and allocation concealment were not explained ^2^ Part of the study was sponsored by the relevant enterprises ^3^ The total sample size is less than OIS ^4^ High heterogeneity | | | | | | |

| **Table s5 Tapentadol compared to placebo for Painful Diabetic Peripheral Neuropathy** | | | | | | |
| --- | --- | --- | --- | --- | --- | --- |
| **Outcomes** | **Illustrative comparative risks* (95% CI)** | | **Relative effect (95% CI)** | **No of Participants (studies)** | **Quality of the evidence (GRADE)** | **Comments** |
|  | Assumed risk | Corresponding risk |  |  |  |  |
|  | **Placebo** | **Tapentadol** |  |  |  |  |
| **studies comparing Tapentadol with placebo for pain outcome** |  | The mean studies comparing tapentadol with placebo for pain outcome in the intervention groups was **0.52 standard deviations lower** (0.93 to 0.11 lower) |  | 730 (3 studies) | ⊕⊝⊝⊝ **very low**^1,2,3^ | SMD -0.52 (-0.93 to -0.11) |
| **studies comparing Tapentadol with placebo for 30% pain reduction** | **Study population** | | **RR 1.25**  (1.07 to 1.45) | 706 (2 studies) | ⊕⊕⊝⊝ **low**^1,3^ |  |
|  | **436 per 1000** | **545 per 1000** (467 to 632) |  |  |  |  |
|  | **Moderate** | |  |  |  |  |
|  | **438 per 1000** | **548 per 1000** (469 to 635) |  |  |  |  |
| **studies comparing Tapentadol with placebo for 50% pain reduction** | **Study population** | | **RR 1.38**  (1.12 to 1.71) | 706 (2 studies) | ⊕⊕⊝⊝ **low**^1,3^ |  |
|  | **282 per 1000** | **389 per 1000** (316 to 482) |  |  |  |  |
|  | **Moderate** | |  |  |  |  |
|  | **283 per 1000** | **391 per 1000** (317 to 484) |  |  |  |  |
| **Effect of Tapentadol on the risk of adverse events** | **Study population** | | **RR 1.33**  (1.2 to 1.49) | 707 (2 studies) | ⊕⊕⊝⊝ **low**^1,3^ |  |
|  | **559 per 1000** | **744 per 1000** (671 to 834) |  |  |  |  |
|  | **Moderate** | |  |  |  |  |
|  | **565 per 1000** | **751 per 1000** (678 to 842) |  |  |  |  |
| GRADE Working Group grades of evidence **High quality:** Further research is very unlikely to change our confidence in the estimate of effect.  **Moderate quality:** Further research is likely to have an important impact on our confidence in the estimate of effect and may change the estimate. **Low quality:** Further research is very likely to have an important impact on our confidence in the estimate of effect and is likely to change the estimate. **Very low quality:** We are very uncertain about the estimate. | | | | | | |
| ^1^ Part of the study was blind, random, and allocative hiding ^2^ High heterogeneity ^3^ Part of the study was sponsored by the relevant enterprises | | | | | | |

| **Table s6 mirogabalin compared to placebo for Painful Diabetic Peripheral Neuropathy** | | | | | | |
| --- | --- | --- | --- | --- | --- | --- |
| **Outcomes** | **Illustrative comparative risks* (95% CI)** | | **Relative effect (95% CI)** | **No of Participants (studies)** | **Quality of the evidence (GRADE)** | **Comments** |
|  | Assumed risk | Corresponding risk |  |  |  |  |
|  | **Placebo** | **Mirogabalin** |  |  |  |  |
| **studies comparing mirogabalin with placebo for pain outcome** |  | The mean studies comparing mirogabalin with placebo for pain outcome in the intervention groups was **0.17 standard deviations lower** (0.29 to 0.05 lower) |  | 1207 (2 studies) | ⊕⊕⊝⊝ **low**^1,2^ | SMD -0.17 (-0.29 to -0.05) |
| **studies comparing mirogabalin with placebo for 50% pain reduction** | **Study population** | | **RR 1.02**  (0.69 to 1.51) | 1207 (2 studies) | ⊕⊝⊝⊝ **very low**^1,2,3,4^ |  |
|  | **249 per 1000** | **254 per 1000** (172 to 376) |  |  |  |  |
|  | **Moderate** | |  |  |  |  |
|  | **305 per 1000** | **311 per 1000** (210 to 461) |  |  |  |  |
| GRADE Working Group grades of evidence **High quality:** Further research is very unlikely to change our confidence in the estimate of effect.  **Moderate quality:** Further research is likely to have an important impact on our confidence in the estimate of effect and may change the estimate. **Low quality:** Further research is very likely to have an important impact on our confidence in the estimate of effect and is likely to change the estimate. **Very low quality:** We are very uncertain about the estimate. | | | | | | |
| ^1^ Selective reporting, allocation hiding not specified ^2^ Part of the study was sponsored by the relevant enterprises ^3^ High heterogeneity ^4^ The 95% confidence interval crosses invalid lines | | | | | | |

| **Table s7 Lacosamide compared to Placebo for Painful Diabetic Peripheral Neuropathy** | | | | | | |
| --- | --- | --- | --- | --- | --- | --- |
| **Outcomes** | **Illustrative comparative risks* (95% CI)** | | **Relative effect (95% CI)** | **No of Participants (studies)** | **Quality of the evidence (GRADE)** | **Comments** |
|  | Assumed risk | Corresponding risk |  |  |  |  |
|  | **Placebo** | **Lacosamide** |  |  |  |  |
| **studies comparing Lacosamide with placebo for pain outcome** |  | The mean studies comparing lacosamide with placebo for pain outcome in the intervention groups was **0.23 standard deviations lower** (0.37 to 0.08 lower) |  | 966 (2 studies) | ⊕⊕⊝⊝ **low**^1,2^ | SMD -0.23 (-0.37 to -0.08) |
| **studies comparing Lacosamide with placebo for 30% pain reduction** | **Study population** | | **RR 1.28**  (1.09 to 1.5) | 966 (2 studies) | ⊕⊕⊝⊝ **low**^1,3^ |  |
|  | **400 per 1000** | **512 per 1000** (436 to 600) |  |  |  |  |
|  | **Moderate** | |  |  |  |  |
|  | **403 per 1000** | **516 per 1000** (439 to 604) |  |  |  |  |
| **Effect of Lacosamide on the risk of adverse events** | **Study population** | | **RR 1.05**  (0.96 to 1.15) | 966 (2 studies) | ⊕⊝⊝⊝ **very low**^2,3^ |  |
|  | **679 per 1000** | **712 per 1000** (651 to 780) |  |  |  |  |
|  | **Moderate** | |  |  |  |  |
|  | **687 per 1000** | **721 per 1000** (660 to 790) |  |  |  |  |
| GRADE Working Group grades of evidence **High quality:** Further research is very unlikely to change our confidence in the estimate of effect.  **Moderate quality:** Further research is likely to have an important impact on our confidence in the estimate of effect and may change the estimate. **Low quality:** Further research is very likely to have an important impact on our confidence in the estimate of effect and is likely to change the estimate. **Very low quality:** We are very uncertain about the estimate. | | | | | | |
| ^1^ Some of the methods of allocation concealment and randomization are not explained ^2^ Part of the study was sponsored by the relevant enterprises ^3^ The 95% confidence interval crosses invalid lines | | | | | | |

| **Table s8 ABT - 894 compared to placebo for Painful Diabetic Peripheral Neuropathy** | | | | | | |
| --- | --- | --- | --- | --- | --- | --- |
| **Outcomes** | **Illustrative comparative risks* (95% CI)** | | **Relative effect (95% CI)** | **No of Participants (studies)** | **Quality of the evidence (GRADE)** | **Comments** |
|  | Assumed risk | Corresponding risk |  |  |  |  |
|  | **Placebo** | **ABT - 894** |  |  |  |  |
| **studies comparing ABT - 894 with placebo for pain outcome** |  | The mean studies comparing mirogabalin with placebo for pain outcome in the intervention groups was **0.04 standard deviations higher** (0.2 lower to 0.27 higher) |  | 342 (2 studies) | ⊕⊕⊕⊝ **moderate**^1^ | SMD 0.04 (-0.2 to 0.27) |
| **studies comparing ABT- 894 with placebo for 30% pain reduction** | **Study population** | | **RR 0.83**  (0.64 to 1.07) | 342 (2 studies) | ⊕⊕⊕⊝ **moderate**^1^ |  |
|  | **482 per 1000** | **400 per 1000** (309 to 516) |  |  |  |  |
|  | **Moderate** | |  |  |  |  |
|  | **487 per 1000** | **404 per 1000** (312 to 521) |  |  |  |  |
| **Effect of ABT- 894 on the risk of adverse events** | **Study population** | | **RR 0.92**  (0.75 to 1.14) | 347 (2 studies) | ⊕⊕⊕⊝ **moderate**^1^ |  |
|  | **552 per 1000** | **508 per 1000** (414 to 629) |  |  |  |  |
|  | **Moderate** | |  |  |  |  |
|  | **560 per 1000** | **515 per 1000** (420 to 638) |  |  |  |  |
| GRADE Working Group grades of evidence **High quality:** Further research is very unlikely to change our confidence in the estimate of effect.  **Moderate quality:** Further research is likely to have an important impact on our confidence in the estimate of effect and may change the estimate. **Low quality:** Further research is very likely to have an important impact on our confidence in the estimate of effect and is likely to change the estimate. **Very low quality:** We are very uncertain about the estimate. | | | | | | |
| ^1^ The 95% confidence interval crosses invalid lines | | | | | | |

| **Table s9 Gabapentin compared to Placebo for Painful Diabetic Peripheral Neuropathy** | | | | | | |
| --- | --- | --- | --- | --- | --- | --- |
| **Outcomes** | **Illustrative comparative risks* (95% CI)** | | **Relative effect (95% CI)** | **No of Participants (studies)** | **Quality of the evidence (GRADE)** | **Comments** |
|  | Assumed risk | Corresponding risk |  |  |  |  |
|  | **Placebo** | **Gabapentin** |  |  |  |  |
| **studies comparing Gabapentin with placebo for pain outcome** |  | The mean studies comparing gabapentin with placebo for pain outcome in the intervention groups was **0.25 standard deviations lower** (0.54 lower to 0.04 higher) |  | 501 (2 studies) | ⊕⊝⊝⊝ **very low**^1,2,3,4^ | SMD -0.25 (-0.54 to 0.04) |
| **studies comparing Gabapentin with placebo for 50% pain reduction** | **Study population** | | **RR 2.39**  (0.57 to 10) | 501 (2 studies) | ⊕⊝⊝⊝ **very low**^1,2,3,4^ |  |
|  | **228 per 1000** | **545 per 1000** (130 to 1000) |  |  |  |  |
|  | **Moderate** | |  |  |  |  |
|  | **185 per 1000** | **442 per 1000** (105 to 1000) |  |  |  |  |
| **Effect of Gabapentin on the risk of adverse events** | **Study population** | | **RR 1.14**  (0.98 to 1.32) | 500 (2 studies) | ⊕⊝⊝⊝ **very low**^1,3,4^ |  |
|  | **582 per 1000** | **664 per 1000** (571 to 769) |  |  |  |  |
|  | **Moderate** | |  |  |  |  |
|  | **529 per 1000** | **603 per 1000** (518 to 698) |  |  |  |  |
| GRADE Working Group grades of evidence **High quality:** Further research is very unlikely to change our confidence in the estimate of effect.  **Moderate quality:** Further research is likely to have an important impact on our confidence in the estimate of effect and may change the estimate. **Low quality:** Further research is very likely to have an important impact on our confidence in the estimate of effect and is likely to change the estimate. **Very low quality:** We are very uncertain about the estimate. | | | | | | |
| ^1^ Some of the methods of allocation concealment and randomization are not explained ^2^ There is some heterogeneity ^3^ The total population is less than OIS ^4^ Part of the study was sponsored by the relevant enterprises | | | | | | |


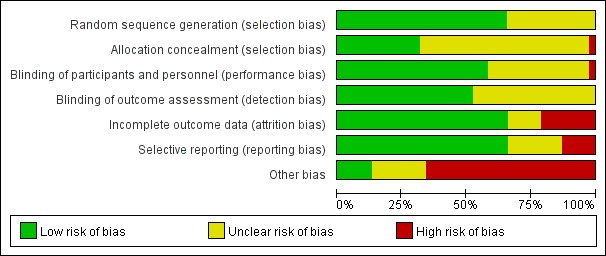


**Figure s1** Risk of bias graph for review authors' judgements about each risk of bias item presented as percentages across all included studies .


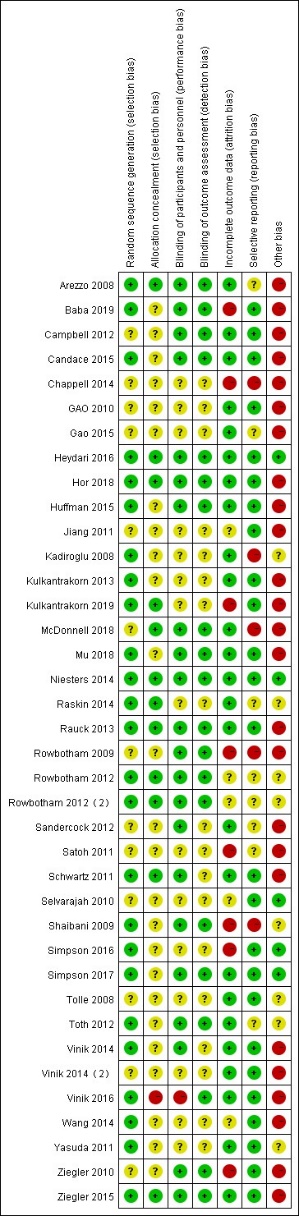


**Figure s2** Risk of bias summary for review authors' judgements about each risk of bias item for each included study.

**
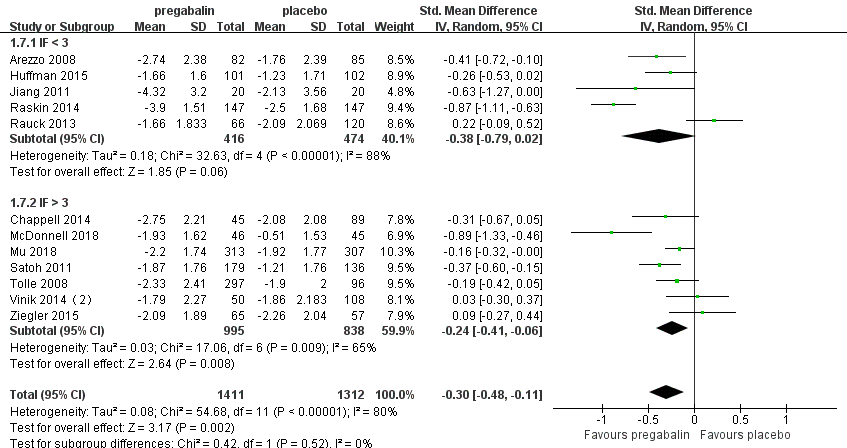
**

**Figure s3** Subgroup analysis of pain score after pregabalin treatment was performed based on dose


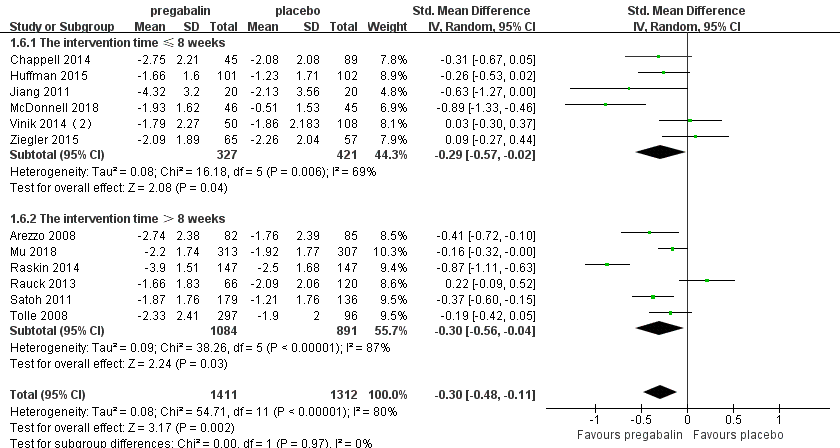


**Figure s4** Subgroup analysis of pain score after pregabalin treatment was performed based on duration of intervention

**
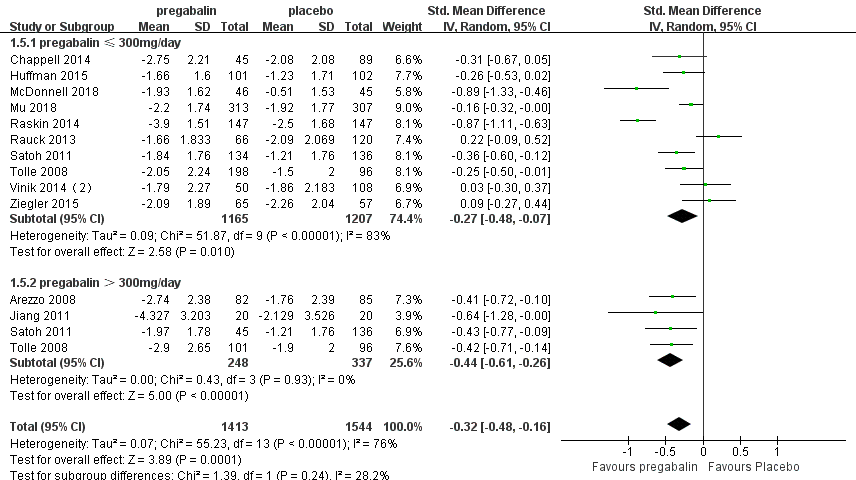
**

**Figure s5** Subgroup analysis of pain score after pregabalin treatment was performed based on quality of the article


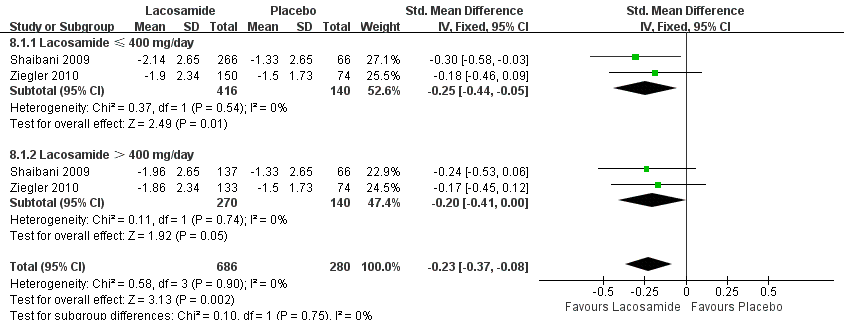


**Figure s6** Subgroup analysis of pain score after Lacosamide treatment was performed based on drug dose


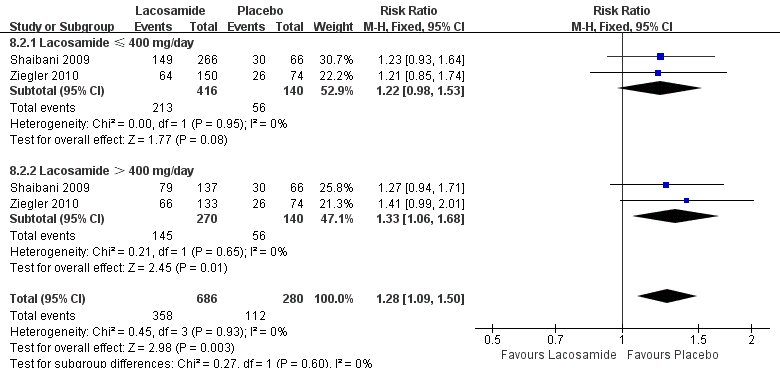


**Figure s7** Subgroup analysis of 30% pain reduction after Lacosamide treatment was performed based on drug dose


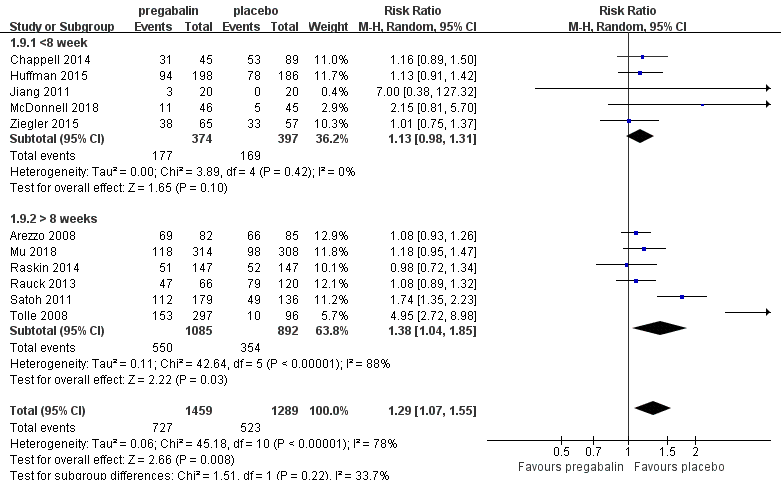


**Figure s8** Subgroup analysis of adverse events after pregabalin treatment was performed based on dose


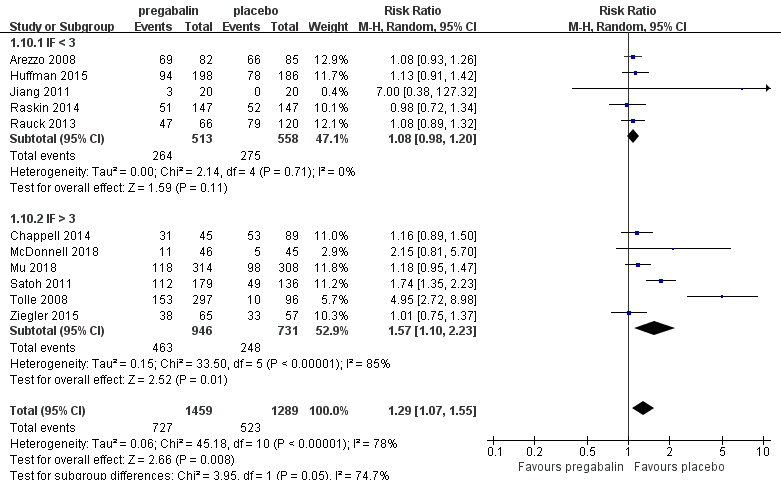


**Figure s9** Subgroup analysis of adverse events after pregabalin treatment was performed based on duration of intervention


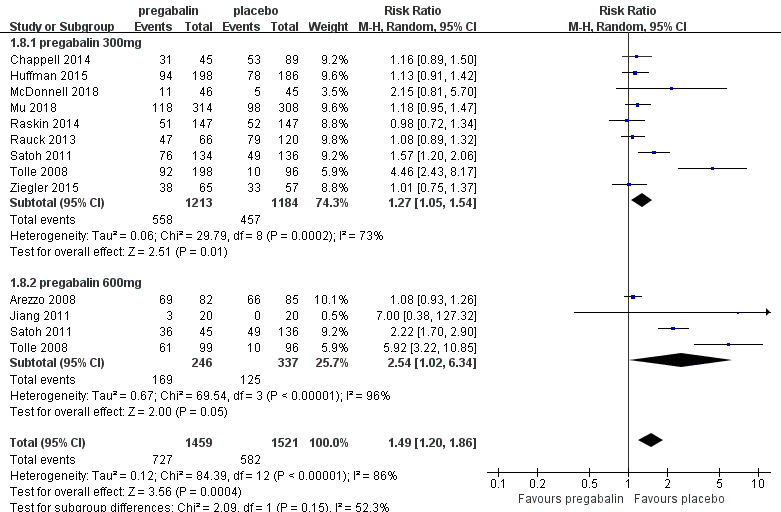


**Figure s10** Subgroup analysis of adverse events after pregabalin treatment was performed based on quality of the article


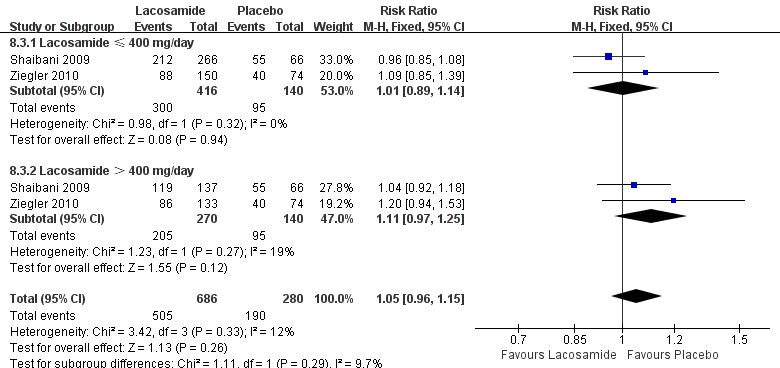


**Figure s11** Subgroup analysis of adverse events after Lacosamide treatment was performed based on drug dosed
